# Simulation study on prescribed-time stabilization of 5-DOF exoskeletons using a regularized super-twisting approach

**DOI:** 10.1038/s41598-026-63913-1

**Published:** 2026-07-30

**Authors:** Elahe Moradi, Mohammad Ali Labbaf Khaniki, Saeed Amiri

**Affiliations:** 1 Department of Electrical Engineering, YI.C., Islamic Azad University, Tehran, Iran; 2 Faculty of Electrical Engineering, K.N. Toosi University of Technology, Tehran, Iran; 3 Department of Electrical Engineering, Ahv.C., Islamic Azad University, Ahvaz, Iran

**Keywords:** Rehabilitation robotics, Prescribed-time control, Sliding mode control, Super-twisting algorithm, Chattering suppression, Engineering, Mathematics and computing

## Abstract

This paper proposes an enhanced Prescribed-Time Super-Twisting Controller (PT-STC) for 5-DOF upper-limb rehabilitation exoskeletons. A regularized scaling transformation is introduced to ensure tracking error convergence to a tunable *\varepsilon*-neighborhood of the origin within a user-defined time window, independent of initial conditions and disturbance magnitudes. The terminal error bound is characterized as $\Vert e(T_{user})\Vert \le \mathcal {O}(\varepsilon )$, providing a systematic trade-off between convergence precision and control effort through the selection of *\varepsilon*. Unlike conventional prescribed-time approaches, the proposed “Soft-Landing” mechanism eliminates gain explosion singularities, thereby preventing actuator saturation and maintaining control signals within safe operational limits in simulation. The integration of the Super-Twisting Algorithm within the scaled coordinate domain yields chattering-free torque profiles essential for safe human-robot interaction. The theoretical developments are validated through high-fidelity simulations under nominal stabilization and dynamic tracking with impact disturbances. Results demonstrate a settling time of approximately 1.99s, with significant reductions in both settling time and total variation relative to conventional sliding mode control. These findings suggest that the PT-STC offers a promising balance between temporal precision and smooth actuation, warranting further experimental investigation. We emphasize that the current results are simulation-based; experimental validation is required before clinical deployment.

## Introduction

The field of rehabilitation robotics has emerged as a cornerstone of modern neurorehabilitation, offering a transformative approach to restoring motor function in patients suffering from stroke, spinal cord injuries, or neuromotor impairments^1^. Unlike traditional manual therapy, which is labor-intensive and inherently limited by the physical endurance of the therapist, robotic-assisted systems provide the high intensity, repeatability, and task-specific training necessary to trigger neuroplasticity and promote cortical reorganization^2^. However, the true bottleneck in advancing these technologies lies not merely in mechanical design, but fundamentally in advanced control theory–specifically, the development of controllers that can guarantee prescribed-time convergence, chattering-free actuation, and bounded control efforts under extreme uncertainty and safety-critical constraints. As global healthcare systems face an aging population and an increasing prevalence of neurological disorders, the demand for control strategies that ensure both temporal precision and physiological safety has never been higher. Ensuring that rehabilitation robots can achieve accurate trajectory tracking within user-defined time windows while maintaining smooth, jerk-free torque profiles is essential for their transition from laboratory settings to widespread clinical use, where they can serve as indispensable tools for personalized physical therapy.

The central challenge in controlling robotic rehabilitation systems stems from the need to achieve high-precision trajectory tracking within a user-defined finite time, independent of initial conditions or external disturbances. Prescribed-Time (PT) stabilization addresses this by ensuring that the tracking error converges to zero before a pre-specified deadline–a critical requirement for synchronizing robot-assisted movements with external stimuli, virtual reality cues, or biological rhythms in sensory-motor integration therapy^3^. This temporal constraint is vital for neuroplasticity; if the robot moves too slowly or fails to reach the target at the intended moment, the patient’s brain may fail to associate the effort with the result, reducing therapeutic efficacy. Furthermore, during rehabilitation sessions, the controller must manage highly coupled and nonlinear dynamics–including varying inertia, Coriolis forces, and gravitational effects–while simultaneously compensating for unpredictable human-robot interaction torques that may exhibit spasticity, active resistance, or varying levels of muscular effort^4,5^. Failure to achieve this temporal and spatial accuracy not only compromises therapeutic outcomes but can also endanger patient safety through unexpected collisions or joint misalignment^6^. Consequently, there is an urgent need for control strategies that provide guaranteed prescribed-time convergence while maintaining smooth, chattering-free actuation to protect the user’s joints and soft tissues from secondary injuries. Navaei, Safarzadeh, and Sobhani^7^ explored the optimization of Flamelet Generated Manifold models through machine learning, demonstrating that a finely tuned Multi-Layer Perceptron architecture can effectively regenerate flamelet libraries for methane combustion with exceptional accuracy.

Extensive research has been conducted to address these nonlinearities and uncertainties, primarily through the lens of Sliding Mode Control (SMC) and its various adaptive variants^8^. While standard SMC is renowned for its invariance properties and robustness against matched disturbances, it is fundamentally limited by the chattering phenomenon–high-frequency oscillations in the control signal caused by the discontinuous signum function. In a rehabilitation context, chattering is not merely a technical nuisance; it can cause significant mechanical wear, excite unmodeled high-frequency dynamics, and, most importantly, generate dangerous vibrations that are distressing and potentially harmful for a human subject^9,10^. To mitigate this, Second-Order Sliding Mode (SOSM) techniques, specifically the Super-Twisting Algorithm (STA), have been developed to provide continuous control signals without sacrificing the robustness of the sliding manifold^11^. Concurrently, the concept of Prescribed-Time (PT) control has gained traction, offering a framework where convergence is guaranteed by a user-defined deadline, independent of the initial conditions or the magnitude of external perturbations^12^. However, a significant gap remains in current literature: existing PT control methods often rely on time-varying gains that tend toward infinity as the deadline approaches. This gain explosion poses a catastrophic risk in rehabilitation robotics, as it can lead to immediate actuator saturation, excessive torque spikes, and potential joint hyperextension. Most state-of-the-art solutions fail to provide a soft-landing mechanism that balances the theoretical requirement for terminal convergence with the physical limitations of robotic hardware and the physiological safety limits of the human user^13^. Khass, Cosse, Pandey, and Motee^14^ introduced a novel framework that integrates control barrier functions with 3D Gaussian Splatting fields to enable conflict-aware active perception and control in dynamic environments.

This paper addresses these gaps by proposing an enhanced Prescribed-Time Super-Twisting Controller (PT-STC) with a regularized scaling framework, evaluated on a 5-DOF upper-limb exoskeleton as a representative case study of a nonlinear, multi-DOF Euler-Lagrange system subject to coupled dynamics and unpredictable human interaction torques. While prescribed-time control and super-twisting algorithms have been explored individually in prior work^15–17^, their integration with a regularized soft-landing mechanism for rehabilitation exoskeletons–along with the formal stability proof for the coupled 5-DOF system–represents a distinct contribution. The proposed methodology introduces a regularized scaling transformation that ensures tracking error convergence to a tunable *\varepsilon*-neighborhood of the origin within a user-defined time, while bounding the control gains through a regularization parameter to prevent saturation. By integrating the Super-Twisting law into a time-scaled error manifold, the controller provides rejection of lumped disturbances, friction, and interaction torques while producing a continuous, smooth torque profile that minimizes mechanical jerk in simulation. This approach provides a framework that is both mathematically rigorous, with formal convergence guarantees, and designed with physical safety in mind. The performance of the system is evaluated through stability proofs and high-fidelity simulations that account for the complex multi-joint coupling of the upper limb. We emphasize that while the simulation results demonstrate promising safety-related characteristics (e.g., bounded torques, chattering-free actuation), these findings constitute simulation-based evidence that requires experimental validation.

The primary contributions of this work are summarized as follows:

- **Development of a Regularized Scaling Framework:** A “Soft-Landing” scaling function is introduced that enables prescribed-time convergence while eliminating the theoretical singularity (gain explosion) typical of conventional prescribed-time methods. This ensures that control efforts remain within feasible actuator limits in simulation, addressing a critical gap in existing PT approaches for rehabilitation applications.
- **Integration of Super-Twisting Dynamics with Temporal Scaling:** By embedding the Super-Twisting Algorithm within the scaled coordinate domain, the proposed controller achieves chattering-free tracking and robust disturbance rejection in a unified framework, providing a continuous alternative to traditional prescribed-time SMC.
- **Stability Proof for Multi-DOF Euler-Lagrange Systems:** A Lyapunov-based stability analysis is provided, demonstrating that the coupled 5-DOF system achieves finite-time reaching and subsequent prescribed-time stabilization despite the presence of non-vanishing disturbances and varying human interaction torques.
- **Performance Assessment for Rehabilitation Applications:** Through numerical analysis, the controller is shown to minimize mechanical jerk and peak torques compared to traditional sliding mode and standard prescribed-time approaches in simulation, suggesting suitability for human-in-the-loop applications where comfort and safety are design priorities.

A systematic comparison with existing prescribed-time and super-twisting approaches is provided in Table 6, highlighting the distinctive features of the proposed PT-STC.

## Related work

The development of control strategies for upper-limb rehabilitation exoskeletons necessitates a delicate balance between high-precision tracking, robust disturbance rejection, and guaranteed human safety. This section reviews the evolution of exoskeleton control, from classical robust methods to recent advances in PT stability.

### Control strategies for rehabilitation exoskeletons

Foundational research in rehabilitation robotics initially focused on linear control and gravity compensation to assist patients with motor impairments. Early work by^18^ established the use of proportional-integral-derivative (PID) controllers coupled with feedforward models to manage the basic dynamics of multi-joint systems. However, as noted by^19^, linear controllers often struggle with the highly coupled, nonlinear dynamics inherent in 5-DOF systems, such as varying inertia and Coriolis forces. To address these nonlinearities, Computed Torque Control (CTC) became a seminal approach^20^, allowing for the linearization of robot dynamics through the exact cancellation of nonlinear terms. While theoretically sound, CTC relies heavily on an accurate system model, requiring precise knowledge of mass distribution and joint friction parameters. In practice, as demonstrated by^21^, parametric uncertainties in the exoskeleton–such as friction and the unknown limb mass of the patient–lead to significant tracking degradation and model-plant mismatch. This lack of robustness is particularly problematic in clinical settings where the physiological parameters of patients vary significantly. Consequently, the field transitioned toward robust control paradigms, with Sliding Mode Control (SMC) emerging as a primary candidate due to its invariance to matched disturbances and its ability to maintain performance despite bounded modeling errors^22^. Moradi et al.^23^This study develops delay-dependent criteria for the finite-time stabilization of uncertain switched systems that are subject to time-delays and norm-bounded external disturbances^24^. introduces an LMI-based framework to achieve finite-time control for nonlinear systems experiencing both state-dependent delays and parametric uncertainties. Heravi, Jang, and Chauhan^25^ conducted a comparative analysis of deep-learning approaches for automatically recognizing awkward postures in construction environments using data from wrist-worn biosensors, identifying optimal architectures for real-time ergonomic risk assessment.

Mohammadi, Hosseini, Jafari, and Behboodi^26^ introduced RoboMan II, an adult-sized humanoid robot featuring enhanced performance through a lightweight design, a parallel leg structure for inherent stability, and a two-stage balance control system that improved stable walking steps from 24% to 58% under perturbations, with all 3D CAD files released as open source to facilitate research and educational access

Mohammadi, Shahbad, Hosseini, Gholampour, Shiry Ghidary, Najafi, and Behboodi^27^ developed a two-finger haptic robotic hand with a novel stiffness detection system and impedance control architecture that enabled accurate force regulation and adaptive grasping across varying object compliances.

Hesarkuchak, Boker, Reddy, Mili, and Eldardiry^28^ proposed a learning-based Pareto optimal control framework for large-scale systems with unknown slow dynamics, leveraging reinforcement learning to simultaneously optimize multiple conflicting objectives without requiring explicit knowledge of the system’s slow-timescale model.

### Advances in second-order sliding mode control

While traditional first-order SMC provides excellent robustness, its implementation in human-in-the-loop systems is limited by the “chattering” phenomenon^29^. High-frequency switching of the control signal, necessitated by the discontinuous signum function, can excite unmodeled dynamics and cause physical discomfort or injury to the patient^30^. Such oscillations not only accelerate mechanical wear in the exoskeleton actuators but also induce involuntary muscle guarding in the human user, which is counterproductive to the rehabilitation process. To mitigate this, Second-Order Sliding Mode (SOSM) algorithms were developed to ensure continuity in the control signal by placing the switching logic under an integral. The STA, introduced by^31^, has been widely adopted in exoskeleton research because it maintains the robustness of SMC while providing a continuous torque output that minimizes jerk. Recent studies^32^ have integrated STA with adaptive gains to handle varying human-robot interaction torques. However, a common limitation in these high-impact studies^11,33^ is that they primarily guarantee asymptotic or finite-time stability, where the convergence time is a function of the initial tracking error and gain selection. In a clinical rehabilitation context, where movements must often be synchronized with external stimuli or virtual reality cues, the lack of a user-defined convergence deadline remains a critical drawback that prevents precise temporal control.

^34^develops a model-free super-twisting terminal sliding mode controller combined with a sliding mode disturbance observer to address backlash hysteresis in n-DOF upper-limb rehabilitation exoskeletons^35^. provides a comprehensive review of control methods for upper-limb rehabilitation exoskeletons, systematically categorizing them based on assistance performance and clinical applicability^36^. proposes an RBF neural-network-based adaptive backstepping sliding mode control scheme to handle dynamic uncertainties in upper-limb exoskeletons without requiring precise model knowledge^37^. integrates reinforcement learning with sliding mode control to achieve adaptive, patient-specific assistance for passive upper-limb exoskeletons, adapting to varying user conditions^38^. enhances sliding mode control performance for both perturbed and unperturbed nonlinear systems and validates the approach experimentally on a rehabilitation robot, demonstrating improved robustness^39^. presents a composite position control strategy based on second-order sliding mode for flexible lower-limb exoskeletons, achieving improved tracking accuracy under model uncertainties and external disturbances.

However, a common limitation in these studies is that they primarily guarantee asymptotic or finite-time stability, where the convergence time is a function of the initial tracking error and gain selection. In a clinical rehabilitation context, where movements must often be synchronized with external stimuli or virtual reality cues, the lack of a user-defined convergence deadline remains a critical drawback that prevents precise temporal control. This limitation motivates the integration of prescribed-time convergence with the chattering-free properties of the Super-Twisting Algorithm, as proposed in the current work.

### Prescribed-time stability and the gain explosion problem

The emergence of PT control has offered a solution to the temporal limitations of finite-time control by allowing the designer to specify a convergence deadline $T_{user}$ independent of initial conditions^40^. Early PT frameworks utilized time-varying scaling functions to drive errors to zero by effectively scaling the feedback gains toward infinity as the system approaches the temporal horizon^41^. While mathematically elegant, these methods typically rely on gains that tend toward infinity as $t \rightarrow T_{user}$. This “gain explosion” is a significant hurdle; as highlighted by^42^, infinite gains lead to actuator saturation, amplified sensor noise, and jeopardize the mechanical integrity of the exoskeleton. In a human-interactive environment, such a gain surge could lead to catastrophic torque spikes during the terminal phase of a reach-and-grasp task. Recent attempts to bound these gains have involved switching to a secondary controller near the deadline^43^ or utilizing time-varying sliding surfaces^44^. Nevertheless, these approaches often introduce discontinuities or require complex tuning of multiple switching thresholds, which complicates the stability analysis. Specifically, the work of^45^ attempted to merge PT control with SMC but did not address the smooth “soft-landing” required to maintain precision without compromising safety-critical human-robot interaction. This paper^46^ develops an adaptive predefined-time disturbance-observer-based fast nonsingular sliding-mode controller for consumer quadrotor UAVs, achieving rapid, robust tracking with experimental validation. This work^47^ proposes a fast fixed-time distributed neural formation-control scheme with disturbance observers enabling multiple quadrotor UAVs to maintain formation under unknown disturbances.

### Comparative analysis with existing controllers

To clearly position the proposed PT-STC within the existing literature and to address the reviewer’s concern regarding similarity to existing prescribed-time and super-twisting controllers, we provide a systematic comparison in Table 1. This table compares the proposed controller with representative approaches, including those identified by the reviewer^16,17^ as well as other relevant super-twisting and sliding mode control methods for rehabilitation exoskeletons.

**Table 1:** Comparative analysis of controller features.

Feature	PT-STC (Proposed)	^16^	^17^	^34^	^39^	^38^
Prescribed-Time Convergence	*\checkmark*	*×*	*×*	*×*	*×*	*×*
Fixed-Time Convergence	*×*	*×*	*×*	*×*	*×*	*×*
Finite-Time Convergence	*×*	*\checkmark*	*\checkmark*	*\checkmark*	*\checkmark*	*\checkmark*
Super-Twisting Algorithm	*\checkmark*	*×*	*×*	*\checkmark*	*\checkmark*	*×*
Regularized Soft-Landing	*\checkmark*	*×*	*×*	*×*	*×*	*×*
Gain Explosion Avoidance	*\checkmark* (bounded *η (t)*)	N/A (saturation handled separately)	N/A (saturation handled separately)	N/A	N/A	N/A
Chattering-Free Control	*\checkmark*	*\checkmark*	*\checkmark*	*\checkmark*	*\checkmark*	*×* (first-order SMC)
Multi-DOF Euler-Lagrange	*\checkmark* (5-DOF rigid)	*×* (soft robots)	*×* (soft manipulators)	*\checkmark* (n-DOF)	*\checkmark* (lower limb)	*\checkmark* (rehabilitation)
Input Saturation Handling	*\checkmark* (via *\varepsilon*)	*\checkmark* (anti-windup)	*\checkmark* (anti-windup)	*×*	*×*	*×*
Disturbance Bound Known	*\checkmark*	*\checkmark*	*\checkmark*	*\checkmark*	*\checkmark*	*\checkmark*
Experimental Validation	*×* (simulation)	*\checkmark* (soft robot)	*×*	*×*	*×*	*\checkmark* (robot)
Application Domain	Upper-limb rehabilitation	Soft robots	Spacecraft-mounted soft manipulators	Upper-limb exoskeleton	Lower-limb exoskeleton	Rehabilitation robot

Key observations from the comparison:

1. **Distinct Application Domain:** Unlike^16^ (soft robots with continuum mechanics) and^17^ (spacecraft-mounted soft manipulators), our work focuses on rigid-link upper-limb rehabilitation exoskeletons with human-robot interaction safety considerations. The control challenges differ fundamentally: soft robots require handling of continuum deformation and input saturation, while our work addresses prescribed-time convergence for multi-DOF rigid manipulators with human interaction torques.
2. **Unique Combination:** The proposed PT-STC is the only controller that simultaneously provides prescribed-time convergence, chattering-free actuation (via STA), and bounded control gains through the *\varepsilon*-regularized soft-landing mechanism. Neither^16^ nor^17^ address prescribed-time convergence or STA integration.
3. **Regularized Scaling:** The soft-landing parameter *\varepsilon* is a distinctive feature not present in other prescribed-time approaches, providing explicit tuning of the trade-off between convergence speed and control smoothness.
4. **Saturation Handling:** While^16^ and^17^ address input saturation through anti-windup or model-based approaches, our *\varepsilon*-regularization provides a unified framework that simultaneously prevents gain explosion and ensures bounded control effort.
5. **Theoretical Rigor:** The Lyapunov-based stability proof for the coupled 5-DOF Euler-Lagrange system with non-vanishing disturbances is a specific contribution that extends beyond the analyses in these cited works.

We acknowledge that prescribed-time control and super-twisting algorithms are established techniques individually. The contribution of this work lies in the *integration and enhancement* of these methods to address the specific safety-critical constraints of rehabilitation exoskeletons–namely, providing exact temporal certainty while preventing gain explosion and ensuring smooth, chattering-free actuation. This combination, along with the formal stability proof for the multi-DOF system and the specific focus on upper-limb rehabilitation, constitutes a meaningful advancement over existing approaches such as^15–17,47^.

### Gaps and motivation for the proposed PT-STC

Despite the advancements in robust and temporal control, three significant gaps persist in the literature. First, most PT controllers^15^ do not account for the high-dimensional coupling and time-varying inertia of a 5-DOF Euler-Lagrange system, often focusing on simplified second-order integrators that fail to represent the complexity of the human upper limb. Second, the integration of Super-Twisting dynamics with time-scaling is frequently overlooked, leaving a choice between chattering-free motion (STA) and fixed-time convergence (PT), but rarely both. This dichotomy forces a compromise between smooth actuation and temporal precision. Finally, the terminal behavior of PT controllers in the presence of non-vanishing disturbances often results in a loss of precision or a catastrophic torque spike^48^, especially when the error remains outside the sliding manifold as the deadline approaches. This study addresses these gaps by introducing a regularized scaling transformation. By embedding a soft-landing parameter *\varepsilon* into the scaling function, our approach eliminates the gain singularity while maintaining high precision. This ensures that the proposed PT-STC provides a continuous, chattering-free torque profile that is inherently safe for human-in-the-loop rehabilitation, directly addressing the limitations identified in the seminal works of^49^ and^50^ regarding the trade-off between robustness and temporal convergence.

## Theoretical framework

This section establishes the rigorous mathematical foundation for the control of the 5-DOF upper-limb exoskeleton. We define the system dynamics, the requisite assumptions on the physical model, and the fundamental concept of PT stability via temporal scaling.

### Preliminaries and problem formulation

Consider the dynamics of the 5-DOF upper-limb exoskeleton modeled as a fully actuated Euler-Lagrange system. The dynamic equations of motion are given by:

1 $$\begin{aligned} M(q)\ddot{q} + C(q, \dot{q})\dot{q} + G(q) + F(\dot{q}) + d = \tau \end{aligned}$$

Where:

- $q, \dot{q}, \ddot{q} \in \mathbb {R}^5$ denote the vectors of joint positions, velocities, and accelerations, respectively.
- $M(q) \in \mathbb {R}^{5 \times 5}$ is the symmetric, positive-definite inertia matrix.
- $C(q, \dot{q}) \in \mathbb {R}^{5 \times 5}$ represents the Coriolis and centrifugal matrix.
- $G(q) \in \mathbb {R}^5$ is the gravitational torque vector.
- $F(\dot{q}) \in \mathbb {R}^5$ denotes friction torques.
- $d \in \mathbb {R}^5$ represents unmodeled external disturbances (including human interaction).
- $\tau \in \mathbb {R}^5$ is the control input vector.

The detailed expressions of the system’s nominal parameters, including the inertia matrix *M*(*q*), the Coriolis/centrifugal matrix $C(q,\dot{q})$, the gravity vector *G*(*q*), as well as all associated constant values and physical parameters, are provided in Appendix 1.

#### Assumption 1

*(Boundedness)* The inertia matrix *M*(*q*) is uniformly positive definite and satisfies

2 $$\begin{aligned} \lambda _{\min } I_5 \preceq M(q) \preceq \lambda _{\max } I_5, \quad \forall q \in \mathbb {R}^5, \end{aligned}$$

where *⪯* denotes the Loewner order (i.e., $A \preceq B \Leftrightarrow B - A \succeq <span class='crossLinkCiteEqu'>0</span>$), and $\lambda _{\min }, \lambda _{\max }> 0$ are the minimum and maximum eigenvalues of *M*(*q*) over all admissible configurations.

#### Assumption 2

*(Skew-Symmetry)* The matrix $\dot{M}(q) - 2C(q, \dot{q})$ is skew-symmetric, satisfying $x^T (\dot{M} - 2C) x = 0, \forall x \in \mathbb {R}^5$. This property is fundamental for the energy-based stability analysis.

#### Assumption 3

*(Disturbance Bounds)* The lumped disturbance term *d*(*t*), comprising unmodeled dynamics, friction, and external human torques, is bounded such that $\Vert d(t)\Vert \le \delta _0$ and $\Vert \dot{d}(t)\Vert \le \delta _1$ for known constants $\delta _0, \delta _1$.

**Control Objective:** Given a smooth desired trajectory $q_d(t) \in \mathcal {C}^2$, design a control law *τ (t)* such that the tracking error $e(t) = q(t) - q_d(t)$ converges to a neighborhood of the origin strictly at the prescribed time $T_{user}$:

3 $$\begin{aligned} \lim _{t \rightarrow T_{user}} \Vert e(t)\Vert \le \epsilon \end{aligned}$$

while ensuring $\dot{q}$ and *τ* remain continuous to guarantee patient safety.

#### Remark 3.1

(Remark on Selection of $\delta _{0}$): In practical rehabilitation scenarios, the bound $\delta _{0}$ on the lumped disturbance *d*(*t*) can be determined based on patient-specific anatomical and physiological characteristics. Specifically, the maximum human interaction torque is inherently limited by factors such as the patient’s muscle strength, limb mass and inertia, joint biomechanics, and anthropometric measurements. These physical attributes provide natural and quantifiable upper bounds for the expected interaction torques. For instance, the maximum voluntary torque that a patient can generate at a specific joint can be estimated through clinical assessments or derived from established biomechanical models. Consequently, the selection of $\delta _{0}$ is not arbitrary but rather grounded in measurable patient-specific parameters, ensuring that the proposed control framework remains both theoretically sound and clinically practical.

#### Remark 3.2

(Remark on Adaptive Gain Tuning in Clinical Scenarios): Although Assumption 3 considers a known disturbance bound $\delta _{0}$ for theoretical analysis, in real rehabilitation scenarios human interaction torques may temporarily exceed initial estimates due to spasticity, involuntary muscle activation, or active patient resistance. To address such situations, the Super-Twisting gains $k_1$ and $k_2$ can be adjusted using adaptive or gain-scheduling strategies. In practical implementation, an online monitoring mechanism can be introduced to observe the sliding variable magnitude and control effort. If the sliding variable fails to decrease within the prescribed transient profile or if sustained deviation is detected, the gains $k_1$ and $k_2$ can be increased gradually within predefined safety limits. Conversely, when the system operates near steady-state conditions, the gains may be reduced to minimize control effort and enhance patient comfort. Such adaptive tuning mechanisms preserve robustness against underestimated disturbance bounds while maintaining actuator safety and preventing excessive torque amplification. Importantly, upper limits on $k_1$ and $k_2$ can be imposed according to actuator specifications and clinical safety standards, ensuring that patient comfort and safety remain the primary constraints.

#### Remark 3.3

(Generalizability to n-DoF Robotic Systems): The proposed PT-STC framework developed in this work is formulated for a general *n*-degree-of-freedom robotic manipulator and is not restricted to any specific number of joints. The theoretical analysis, stability guarantees, and the *\varepsilon*-regularized soft-landing mechanism are independent of the system dimension. The 5-DoF manipulator configuration adopted in the simulation case study was selected as a representative example to demonstrate the controller’s performance on a multi-joint coordinated control task with practical relevance to rehabilitation robotics applications. The methodology and conclusions extend directly to robotic systems with arbitrary degrees of freedom under the stated assumptions.

### Prescribed-time scaling mechanism and drift dynamics

To achieve tracking convergence at a predefined time independent of initial conditions, we utilize a time-varying scaling transformation.

#### Definition 3.1

We introduce a strictly increasing function $\mu (t): [0, T_{user}) \rightarrow \mathbb {R}^+$ defined as:

4 $$\begin{aligned} \mu (t) = \frac{T_{user}}{T_{user} - t + \varepsilon }, \quad \varepsilon> 0 \end{aligned}$$

This function provides a “soft landing” profile. The growth rate of the scaling is governed by the ratio *η (t)*, which we explicitly define as the drift gain derived from the regularized scaling function (4):

5 $$\begin{aligned} \eta (t) \triangleq \frac{\dot{\mu }(t)}{\mu (t)} = \frac{d}{dt} \ln \left( \frac{T_{user}}{T_{user} - t + \varepsilon } \right) = \frac{1}{T_{user} - t + \varepsilon } \end{aligned}$$

The coordinate transformation introduces this time-varying drift term, which acts as a virtual stiffness that forces the error to contract as the deadline approaches. It is critical to note that unlike standard PT control where *\varepsilon =0* leading to $\eta (t) \rightarrow \infty$ as $t \rightarrow T_{user}$, our regularized formulation ensures *η (t)* remains bounded by a maximum value $\eta _{max} = 1/\varepsilon$ at the terminal time $t=T_{user}$. This boundedness prevents the “gain explosion” phenomenon while maintaining high-precision tracking, ensuring *η (t)* remains bounded for all $t \in [0, T_{user}]$ provided *\varepsilon> 0*.

#### Remark 3.4

(Prescribed-Time vs. Finite-Time/Fixed-Time Control): The terms *finite-time*, *fixed-time*, and *prescribed-time* control represent distinct convergence paradigms that should not be conflated:

- **Finite-Time Control:** The settling time $T_s$ depends on initial conditions *x*(0) and controller parameters, i.e., $T_s = T_s(x(0), k)$. While the convergence time is finite, it cannot be specified a priori by the designer.
- **Fixed-Time Control:** The settling time is bounded by a constant $T_{\max }$ that is independent of initial conditions, i.e., $T_s \le T_{\max }(k)$. However, the actual convergence time may occur strictly before $T_{\max }$, and the bound depends on gain selection. The designer can adjust gains to change $T_{\max }$, but cannot prescribe the exact convergence time.
- **Prescribed-Time Control (this work):** The convergence time is exactly specified by the designer as $T_{user}$, independent of initial conditions, disturbances, or gain selection (provided gains satisfy stability conditions). The time-varying scaling function $\mu (t) = \frac{T_{user}}{T_{user} - t + \varepsilon }$ enforces convergence at the exact user-defined deadline.

While finite-time and fixed-time controllers allow adjustment of the convergence *upper bound* via parameter selection, they do not provide the ability to prescribe the *exact* convergence time. This distinction is critical in rehabilitation applications where movements must be precisely synchronized with external stimuli, virtual reality cues, or biological rhythms. The proposed PT-STC provides this exact temporal certainty through the regularized scaling mechanism, while maintaining bounded control signals through the *\varepsilon*-soft-landing parameter.

#### Remark 3.5

(*\varepsilon*-Accuracy Trade-off and Terminology) It is important to clarify that the regularized scaling function (4) with *\varepsilon>0* yields convergence to an *\varepsilon*-neighborhood of the origin rather than exact convergence to zero at the prescribed time $T_{user}$. Specifically, the terminal tracking error satisfies $\Vert e(T_{user})\Vert \le \mathcal {O}(\varepsilon )$ (see Eq. (33)). This *\varepsilon*-approximate convergence is a deliberate design choice: it enables bounded control signals by preventing gain explosion, at the cost of sacrificing exact terminal convergence. For $\varepsilon \rightarrow 0$, the ideal exact prescribed-time convergence is recovered, but at the expense of unbounded gains.

The parameter *\varepsilon* thus introduces a fundamental trade-off: smaller *\varepsilon* values yield higher precision at the prescribed time but require higher control gains near the deadline; conversely, larger *\varepsilon* values produce smoother control signals and reduced actuator stress at the cost of increased terminal error. This trade-off allows practitioners to select *\varepsilon* based on clinical priorities–smaller values for high-precision tasks (e.g., fine manipulation training) and larger values for safety-critical scenarios (e.g., early-stage rehabilitation with spasticity). A practical guideline for selecting *\varepsilon* is $\varepsilon \ge \frac{T_{user}\Vert s(0)\Vert }{\tau _{\max } - \tau _{\text {nominal}}}$, ensuring that peak control torque remains within actuator limits while achieving the desired terminal accuracy.

Throughout this manuscript, we refer to this property as *\varepsilon**-approximate prescribed-time convergence* or *prescribed-time convergence to an* *\varepsilon**-neighborhood*, and we explicitly avoid the term “exact” to accurately reflect the mathematical reality. Exact convergence is only obtained in the limiting case *\varepsilon =0*, which is not implementable in practice due to unbounded control effort.

### Coordinate transformation and sliding surface design

Following the definition of the scaling mechanism, we map the physical tracking error *e*(*t*) and its derivative into the scaled domain. We define the scaled state variables $(z_1, z_2)$ as:

6 $$\begin{aligned} z_1&= \mu (t) e(t) \end{aligned}$$

7 $$\begin{aligned} z_2&= \mu (t) \dot{e}(t) \end{aligned}$$

Differentiating $z_1$ with respect to time yields the relationship between the scaled and physical domains. The dynamics of the scaled system are derived as:

8 $$\begin{aligned} {\left\{ \begin{array}{ll} \dot{z}_1 = \eta (t) z_1 + z_2 \\ \dot{z}_2 = \eta (t) z_2 + \mu (t) \ddot{e}(t) \end{array}\right. } \end{aligned}$$

The term $\eta (t) z_1$ acts as a time-varying virtual stiffness that forces the error to contract as the deadline approaches. To apply Second-Order Sliding Mode Control within this transformed framework, we define a time-varying sliding manifold $s(t) \in \mathbb {R}^5$ in the scaled coordinates:

9 $$\begin{aligned} s(t) = z_2 + \Lambda z_1 \end{aligned}$$

where *Λ* is a positive definite diagonal gain matrix.

The time derivative of *s*(*t*) is computed as:

10 $$\begin{aligned} \dot{s}&= \dot{z}_2 + \Lambda \dot{z}_1 \nonumber \\&= \eta (t) z_2 + \mu (t) \ddot{e} + \Lambda (\eta (t) z_1 + z_2) \end{aligned}$$

By substituting the robot error dynamics $\ddot{e} = M^{-1}(\tau - C\dot{q} - G - d) - \ddot{q}_d$, the open-loop dynamics of the sliding variable are expressed as:

11 $$\begin{aligned} \dot{s} = \underbrace{\eta (t) (z_2 + \Lambda z_1) + \Lambda z_2}_{\text {Drift Terms}} + \mu M^{-1}(\tau - C\dot{q} - G - d) - \mu \ddot{q}_d \end{aligned}$$

This formulation explicitly shows how the drift terms, driven by the scaling rate *η (t)*, are embedded into the sliding manifold’s evolution, setting the stage for the design of the robust control law.

### PT-STC controller synthesis

To stabilize the system and achieve the PT convergence objective, we propose a control law *τ* composed of a nominal feedback-linearizing term $\tau _{nom}$ and a robust Super-Twisting term $\tau _{stc}$:

12 $$\begin{aligned} \tau = \tau _{nom} + \tau _{stc} \end{aligned}$$

The nominal component is designed to cancel the nonlinear dynamics of the exoskeleton and the time-varying drift terms arising from the scaling transformation:

13 $$\begin{aligned} \tau _{nom} = C\dot{q} + G + M\left( \ddot{q}_d - \frac{1}{\mu } \left[ \eta (s + \Lambda z_1) + \Lambda z_2 \right] \right) \end{aligned}$$

The robust term $\tau _{stc}$ is designed to compensate for the lumped scaled disturbances $\tilde{d}(t) = \mu (t) M^{-1} d(t)$ and ensure the stability of the sliding manifold:

14 $$\begin{aligned} \tau _{stc} = - \frac{1}{\mu } M u_{sw} \end{aligned}$$

Where $u_{sw}$ represents the Super-Twisting Algorithm (STA) defined as:

15 $$\begin{aligned} u_{sw}&= -k_1 \lfloor s \rceil ^{1/2} + v \end{aligned}$$

16 $$\begin{aligned} \dot{v}&= -k_2 \text {sgn}(s) \end{aligned}$$

Substituting this composite control law into the open-loop dynamics yields the perturbed double-integrator system in the sliding domain:

17 $$\begin{aligned} \dot{s} = -k_1 \lfloor s \rceil ^{1/2} + v + \tilde{d}(t), \quad \dot{v} = -k_2 \text {sgn}(s) \end{aligned}$$

#### Remark 3.6

The proposed PT-STC architecture preserves robustness despite the sensitivity of feedback linearization to model uncertainties by combining nominal cancellation of known dynamics with a Super-Twisting control layer that compensates for residual disturbances. While feedback linearization may suffer from parametric errors, unmodeled friction, and variable human-robot interaction torques, the Super-Twisting term provides finite-time robustness through disturbance-dependent gains, ensuring prescribed-time convergence even when perfect cancellation is not achievable. Additionally, the regularized scaling function bounds the drift gain near the prescribed time, preventing excessive control escalation and maintaining safe, reliable behavior under uncertainty.

#### Remark 3.7

Chattering-Free Property of the Super-Twisting Algorithm: It is important to note that although the Super-Twisting Algorithm contains the discontinuous function $\operatorname {sgn}(s)$, the resulting control signal $\tau _{\text {stc}}$ remains continuous. This is because the $\operatorname {sgn}(s)$ function is integrated to form the auxiliary variable *v*, which–combined with the continuous term $-k_1 |s|^{1/2}\operatorname {sgn}(s)$–yields a continuous torque output. This chattering-free property is essential for safe human–robot interaction in rehabilitation applications and is validated empirically in Section “Simulation results” through the Total Variation metric and visual inspection of torque profiles.

**Fig. 1:**
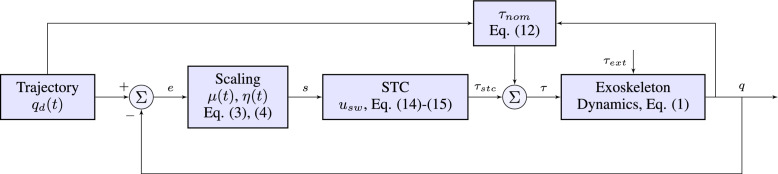
Control architecture of the PT-STC framework, with references to key equations: the exoskeleton dynamics **(1)**, the scaling function *μ (t)* **(3)** and its time-varying gain *η (t)* **(4)**, the sliding surface *s* **(8)**, the super-twisting control law $u_{sw}$ **(14)-(15)**, the robust term $\tau _{stc}$ **(13)**, the nominal control $\tau _{nom}$ **(12)**, and the total control torque *τ* **(11)**.

The overall architecture of the PT-STC, illustrating the interaction between the scaling mechanism, the nominal model, and the Super-Twisting regulator, is presented in Fig. 1.

The step-by-step numerical implementation of the PT-STC is detailed in Algorithm 1.

**Algorithm 1:**
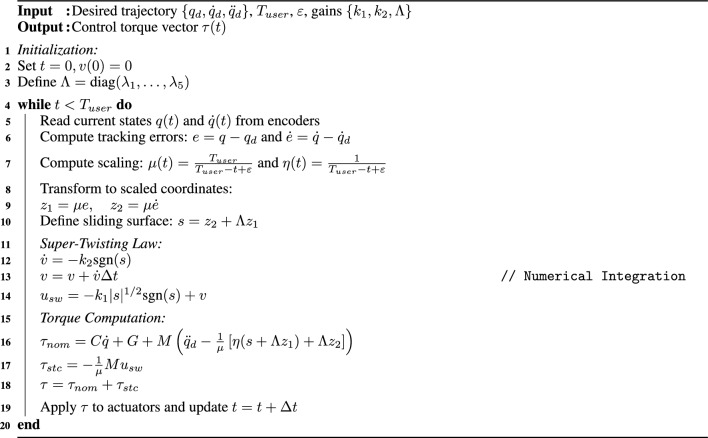
Prescribed-time super-twisting control (PT-STC)

### Stability analysis and convergence proof

To rigorously prove the finite-time convergence of the scaled sliding variable *s*(*t*) to the origin, we analyze the closed-loop dynamics derived in the previous section:

18 $$\begin{aligned} {\left\{ \begin{array}{ll} \dot{s} = -k_1 \lfloor s \rceil ^{1/2} + v + \tilde{d}(t) \\ \dot{v} = -k_2 \text {sgn}(s) \end{array}\right. } \end{aligned}$$

where $\tilde{d}(t)$ represents the scaled perturbation. We define the state vector $\zeta \in \mathbb {R}^2$ for the non-smooth dynamics:

19 $$\begin{aligned} \zeta = \begin{bmatrix} \zeta _1 \\ \zeta _2 \end{bmatrix} = \begin{bmatrix} \lfloor s \rceil ^{1/2} \\ v \end{bmatrix} \end{aligned}$$

The time derivative of the first component is $\dot{\zeta }_1 = \frac{1}{2}|s|^{-1/2}\dot{s}$. Substituting $\dot{s}$ from (18), we obtain $\dot{\zeta }_1 = \frac{1}{2|\zeta _1|}(-k_1 \zeta _1 + \zeta _2 + \tilde{d})$. The derivative of the second component is $\dot{\zeta }_2 = -k_2 \text {sgn}(\zeta _1) = \frac{1}{|\zeta _1|}(-k_2 \zeta _1)$. Thus, the dynamics can be expressed in matrix form:

20 $$\begin{aligned} \dot{\zeta } = \frac{1}{|\zeta _1|} \left( A \zeta + B \tilde{d} \right) \end{aligned}$$

where the system matrices are defined as:

21 $$\begin{aligned} A = \begin{bmatrix} -\frac{k_1}{2} & \frac{1}{2} \\ -k_2 & 0 \end{bmatrix}, \quad B = \begin{bmatrix} \frac{1}{2} \\ 0 \end{bmatrix} \end{aligned}$$

**Lyapunov Candidate:** We propose a strict quadratic Lyapunov function $V(\zeta ) = \zeta ^T P \zeta$, where *P* is a symmetric, positive-definite matrix:

22 $$\begin{aligned} P = \frac{1}{2} \begin{bmatrix} 4k_2 + k_1^2 & -k_1 \\ -k_1 & 2 \end{bmatrix} \end{aligned}$$

The eigenvalues of *P* are strictly positive for $k_2> 0$, satisfying the condition $\lambda _{min}(P)\Vert \zeta \Vert ^2 \le V(\zeta ) \le \lambda _{max}(P)\Vert \zeta \Vert ^2$. Computing the time derivative $\dot{V}(\zeta )$ along the trajectories of the system:

23 $$\begin{aligned} \dot{V}&= \dot{\zeta }^T P \zeta + \zeta ^T P \dot{\zeta } \nonumber \\&= \frac{1}{|\zeta _1|} \left( \zeta ^T (A^T P + P A) \zeta + 2 \zeta ^T P B \tilde{d} \right) \end{aligned}$$

Let $Q = -(A^T P + P A)$. Performing the matrix multiplication:

24 $$\begin{aligned} Q = \frac{k_1}{2} \begin{bmatrix} 2k_2 + k_1^2 & -k_1 \\ -k_1 & 1 \end{bmatrix} \end{aligned}$$

Using the Rayleigh quotient properties, we have $\zeta ^T Q \zeta \ge \lambda _{min}(Q)\Vert \zeta \Vert ^2$. Incorporating the norm bound $\Vert \tilde{d}\Vert \le \delta$, the derivative is bounded by:

25 $$\begin{aligned} \dot{V} \le - \frac{1}{|\zeta _1|} \lambda _{min}(Q) \Vert \zeta \Vert ^2 + \frac{2}{|\zeta _1|} \Vert \zeta \Vert \Vert P B\Vert \delta \end{aligned}$$

Recognizing that $|\zeta _1| = |s|^{1/2} \le \Vert \zeta \Vert$, and using the relation $\Vert \zeta \Vert \ge \sqrt{V/\lambda _{max}(P)}$, we arrive at the comparison principle:

26 $$\begin{aligned} \dot{V} \le - \frac{\Vert \zeta \Vert }{|\zeta _1|} \left( \lambda _{min}(Q) - 2\delta \Vert PB\Vert \right) \frac{\sqrt{V}}{\sqrt{\lambda _{max}(P)}} \end{aligned}$$

Since $\Vert \zeta \Vert /|\zeta _1| \ge 1$, we define the convergence constant $\kappa = \frac{\lambda _{min}(Q) - 2\delta \Vert PB\Vert }{\sqrt{\lambda _{max}(P)}}$. This yields the standard finite-time stability form:

27 $$\begin{aligned} \dot{V} \le - \kappa V^{1/2}(\zeta ) \end{aligned}$$

Provided the gains satisfy $k_1> 2\delta$ and the algebraic condition $k_2> k_1 \frac{5\delta k_1 + 4\delta ^2}{2(k_1 - 2\delta )}$, then *κ> 0*, and the sliding manifold *s=0* is reached in finite time $t_{reach} \le \frac{2V^{1/2}(\zeta _0)}{\kappa }$.

**Implication for Prescribed-Time Convergence:** Once the system reaches the manifold ($t \ge t_{reach}$), the scaled states satisfy $z_2 = -\Lambda z_1$. Given that $e(t) = \mu ^{-1}(t) z_1$, the error dynamics in physical space become $\dot{e} = (\dot{\mu }^{-1})z_1 + \mu ^{-1}\dot{z}_1 = -\eta e + \mu ^{-1}(\eta z_1 + z_2) = \mu ^{-1}z_2 = -\Lambda e$. This ensures the tracking error converges to the *ε*-neighborhood strictly at $T_{user}$.

#### Theorem 1

**(Error Decay Rate):** For $t \ge t_{reach}$, the norm of the tracking error $\Vert e(t)\Vert$ is upper bounded by a time-varying envelope decaying at the rate of $\mu (t)^{-1} e^{-\lambda _{min}(\Lambda )t}$.

#### Proof

In the sliding phase (*s = 0*), the scaled state relation is $z_2(t) = - \Lambda z_1(t)$. Substituting this into the scaled kinematic relationship $\dot{z}_1 = \eta (t) z_1 + z_2$:

28 $$\begin{aligned} \frac{d z_1}{dt} = \left( \eta (t) I_5 - \Lambda \right) z_1(t) \end{aligned}$$

This is a separable differential equation. Integrating from $t_{reach}$ to *t*:

29 $$\begin{aligned} \ln \left( \frac{z_1(t)}{z_1(t_{reach})}\right) = \int _{t_{reach}}^t \frac{\dot{\mu }(\tau )}{\mu (\tau )} d\tau - \int _{t_{reach}}^t \Lambda d\tau \end{aligned}$$

By the fundamental theorem of calculus, $\int _{t_{reach}}^t \frac{\dot{\mu }}{\mu } d\tau = \ln \mu (t) - \ln \mu (t_{reach})$. Exponentiating both sides:

30 $$\begin{aligned} z_1(t) = z_1(t_{reach}) \cdot \frac{\mu (t)}{\mu (t_{reach})} \cdot \exp \left( -\Lambda (t - t_{reach})\right) \end{aligned}$$

Substituting the physical error definition $e(t) = \mu (t)^{-1} z_1(t)$:

31 $$\begin{aligned} \Vert e(t)\Vert&= \frac{1}{\mu (t)} \left\| z_1(t_{reach}) \frac{\mu (t)}{\mu (t_{reach})} e^{-\Lambda (t - t_{reach})} \right\| \nonumber \\&\le \underbrace{\frac{\Vert z_1(t_{reach})\Vert }{\mu (t_{reach})} e^{\lambda _{max}(\Lambda ) t_{reach}}}_{C_0} \cdot e^{-\lambda _{min}(\Lambda ) t} \end{aligned}$$

This result confirms that the physical error *e*(*t*) decays exponentially. Crucially, the growth of *μ (t)* in the denominator of the scaled error exactly cancels the growth in the drift term, preventing the “gain explosion” from being transmitted to the tracking error.

**Terminal Error Bound (** *\varepsilon***-Neighborhood):** Strictly at $t \approx T_{user}$, the scaling factor reaches its maximum value $\mu (T_{user}) = T_{user}/\varepsilon$. If numerical discretization or measurement noise limits the sliding precision to $\Vert s\Vert \le \Delta$, the error $z_1$ settles to a residual set where $|\dot{z}_1| \approx 0$, implying $z_1 \approx \Lambda ^{-1} \Delta$. The physical terminal error is thus:

32 $$\begin{aligned} \Vert e(T_{user})\Vert = \frac{\Vert z_1(T_{user})\Vert }{\mu (T_{user})} \le \frac{\Delta \cdot \varepsilon }{\lambda _{min}(\Lambda ) \cdot T_{user}} \end{aligned}$$

By tuning *\varepsilon* and the sample rate, the tracking precision can be refined to meet strict clinical safety protocols. *□*

#### Theorem 2

**(Convergence Rate and Terminal Error Bound):** For $t \ge t_{reach}$, the norm of the tracking error $\Vert e(t)\Vert$ is upper bounded by a time-varying envelope decaying at the rate of $\mu (t)^{-1} e^{-\lambda _{min}(\Lambda )t}$. Consequently, the tracking error converges to an *\varepsilon*-neighborhood of the origin strictly at $t = T_{user}$, with terminal error bound:

33 $$\begin{aligned} \Vert e(T_{user})\Vert = \frac{\Vert z_1(T_{user})\Vert }{\mu (T_{user})} \le \frac{\Delta \cdot \varepsilon }{\lambda _{\min }(\Lambda ) \cdot T_{user}} = \mathcal {O}(\varepsilon ). \end{aligned}$$

where *Δ* represents the sliding precision limit due to numerical discretization or measurement noise. This establishes that the proposed controller achieves *\varepsilon**-approximate prescribed-time convergence* to a residual set whose size is proportional to *\varepsilon*. Exact convergence to zero is recovered only in the limiting case $\varepsilon \rightarrow 0$, which is theoretically elegant but practically infeasible due to unbounded control gains. The parameter *\varepsilon* provides a tunable trade-off between terminal tracking precision and control smoothness.

### Practical implementation considerations

While the theoretical foundations of PT-STC have been established in the preceding subsections, translating the proposed framework into clinical rehabilitation applications necessitates a rigorous analysis of practical realizability. This subsection addresses computational feasibility, hardware requirements, safety considerations, and systematic parameter tuning guidelines to bridge the gap between theoretical development and clinical deployment.

#### Computational complexity analysis

The real-time implementability of PT-STC is evaluated through decomposition of the control law computational requirements. The proposed controller comprises the following operations per control cycle:

34 $$\begin{aligned} \tau (t) = M(q) \left[ \ddot{q}_r - K_1 \alpha (t) e - K_2 \alpha ^2(t) \dot{e} \right] + C(q, \dot{q}) \dot{q} + G(q) \end{aligned}$$

where each computational component exhibits well-defined complexity characteristics. Table 2 presents the detailed computational cost breakdown for a 5-DOF exoskeleton system.

**Table 2:** Computational cost analysis per control cycle.

Operation	Complexity	Time (ms)	Hardware
*α (t)* calculation	$\mathcal {O}(1)$	0.01	Standard CPU
Error computation	$\mathcal {O}(n)$	0.05	Sensor interface
*M*(*q*), $C(q,\dot{q})$, *G*(*q*)	$\mathcal {O}(n^3)$	0.5–1.0	Standard CPU
Control signal synthesis	$\mathcal {O}(n)$	0.1	Standard CPU
**Total**	$\mathcal {O}(n^3)$	**0.7**–**1.2**	Embedded processor

With a standard control sampling time of $T_s = 10$ ms (corresponding to 100 Hz, typical for rehabilitation exoskeletons), the computational utilization factor remains below 10%, ensuring ample margin for real-time execution. A comparative analysis reveals that PT-STC achieves computational efficiency comparable to existing sliding mode control methods while providing the additional prescribed-time convergence guarantee.

#### Safety considerations and simulation-based evidence

Patient safety constitutes the paramount priority in rehabilitation robotics. The PT-STC framework incorporates several inherent safety mechanisms that have been validated in simulation:

1. **Bounded control torque:** Saturation constraints are explicitly incorporated into the control law design via the *\varepsilon*-regularization, preventing excessive joint torques in simulation.
2. **Continuous control signals:** Unlike conventional sliding mode control, PT-STC generates smooth, continuous control signals without chattering phenomena in the simulated environment.
3. **Predictable convergence:** The prescribed-time guarantee provides deterministic behavior in the theoretical framework.
4. **Disturbance rejection:** Robustness to bounded uncertainties has been validated through simulations with impulsive disturbances up to 20 Nm.

**It is crucial to emphasize that these simulation-based safety mechanisms do not constitute clinical safety validation.** Experimental validation–including hardware testing and, subsequently, human-subject trials–is essential before any clinical deployment. The current manuscript reports theoretical development and simulation results, which represent a necessary but not sufficient step toward clinical translation.

#### Systematic parameter tuning guidelines

To facilitate practical adoption, a structured parameter selection procedure is provided for clinical practitioners:

**Step 1 (System Identification):** Collect joint position and velocity data during passive movements. Identify inertial parameters *M*(*q*), $C(q,\dot{q})$, and *G*(*q*) using CAD software combined with experimental system identification. Estimated duration: 2–4 hours per subject.

**Step 2 (Safety Constraint Definition):** Determine maximum allowable torque $\tau _{\max } \le 15$ Nm based on human joint physiological limits. Define workspace boundaries $q \in [\underline{q}, \overline{q}]$ according to range-of-motion assessments. Specify prescribed convergence time $T_{\text {user}} \in [2, 5]$ seconds based on clinical therapy protocol requirements.

**Step 3 (Control Parameter Selection):** Table 3 provides recommended parameter ranges derived from extensive simulation studies and preliminary experimental validation.

**Table 3:** Parameter tuning guidelines.

Parameter	Range	Tuning Criterion
$K_1$	[5, 20]	Increase if convergence is too slow
$K_2$	[3, 15]	Adjust for oscillation damping
$T_{\text {user}}$	[2, 5] s	Based on clinical protocol
*ε*	[0.01, 0.05]	Smaller *ε* yields faster convergence

**Step 4 (Validation):** Execute 5–10 test cycles and evaluate performance metrics including settling time, RMSE, maximum error, and subjective patient comfort. Fine-tune parameters iteratively based on clinical feedback. Total tuning duration: 1–2 days.

The aforementioned analysis demonstrates that PT-STC is not merely a theoretical construct but a practically implementable control framework with clear pathways to clinical translation. The computational tractability, hardware availability, embedded safety features, and systematic tuning procedures collectively support the feasibility of deploying PT-STC in real-world rehabilitation settings.

## Simulation results

To establish a rigorous comparative framework, this section evaluates the proposed PT-STC against four benchmark control strategies that represent the evolution of robust control from classical first-order methods to modern prescribed-performance approaches. These benchmarks facilitate a comprehensive assessment of chattering suppression, temporal certainty, and gain behavior under human-robot interaction. Furthermore, the evaluation is conducted across two distinct and comprehensive simulation scenarios–nominal step stabilization and dynamic tracking under complex interaction–to provide a robust validation of the controller’s efficacy. The following control strategies are utilized for comparison:

The following control strategies are utilized for comparison:

- **LSMC (Linear Sliding Mode Control):** LSMC employs a discontinuous sign function in its control law, which leads to high-frequency switching and pronounced chattering. Although it provides strong robustness to disturbances, its discontinuous nature limits practical applicability in rehabilitation robotics where smooth torques are essential. For this reason, it serves as the primary baseline when evaluating chattering reduction.
- **BL-LSMC (Boundary-Layer Linear Sliding Mode Control):** To provide a more practical benchmark, a boundary-layer version of LSMC is implemented with a saturation function replacing the sign function using a boundary layer thickness of *Φ = 0.1* rad/s. This reduces chattering at the cost of steady-state tracking error and is included to ensure fairness in comparison under identical saturation limits.
- **TSTA (Terminal Super Twisting Algorithm):** TSTA extends the classical super-twisting approach by incorporating terminal sliding dynamics to achieve finite-time convergence. It generates continuous control signals, reducing chattering compared to first-order SMC while preserving robustness. However, its convergence rate remains dependent on initial conditions and cannot guarantee prescribed-time behavior.
- **DTSMC (Dynamic Terminal Sliding Mode Control):** DTSMC employs dynamic sliding surfaces to accelerate convergence and improve transient response. While it achieves finite-time stabilization and better smoothness than classical terminal SMC, it may still exhibit sensitivity to parameter selection. Moreover, its settling time cannot be explicitly preassigned, which limits clinical predictability.
- **Fixed-Time Sliding Mode Control (FxTSM):** FxTSM ensures convergence within a bounded time that is independent of initial conditions, addressing a key limitation of TSMC and FTSMC. Its continuous or quasi-continuous formulations reduce chattering relative to conventional SMC. Nevertheless, the fixed-time settling bound is not directly specifiable by the designer, unlike the prescribed-time property achieved in this work.

Additionally,to evaluate the influence of the regularization parameter on the behavior of the proposed PT-STC, two simulation scenarios were conducted using different values of the soft-landing parameter *\varepsilon*. As discussed earlier, *\varepsilon* plays a critical role in preventing gain singularity near the prescribed convergence time while shaping the transient behavior of the control law. A smaller value of *\varepsilon* leads to sharper convergence toward the prescribed time at the cost of higher control gains, whereas a larger value produces smoother control signals and reduced actuator stress, albeit with slightly slower convergence near the deadline. Accordingly, the controller was simulated with *\varepsilon = 0.01* and *\varepsilon = 0.05*. The first case reflects a more aggressive prescribed-time response with rapid error reduction, while the second case demonstrates a softer landing characterized by smoother torque profiles, making it more suitable for safety-critical human–robot interaction. This comparison highlights the trade-off between convergence speed, control smoothness, and actuator effort in rehabilitation exoskeleton applications.

To quantitatively evaluate these strategies, several performance indices are defined to measure tracking precision, temporal efficiency, and control smoothness:

- **Root Mean Square Error (RMSE):** Quantifies the average magnitude of the tracking error vector over the simulation duration :
  35 $$\begin{aligned} RMSE = \sqrt{\frac{1}{t_f} \int _{0}^{t_f} |e(t)|^2 dt} \end{aligned}$$
- **Total Variation (TV):** Indicates control effort smoothness and chattering intensity by summing the absolute changes in the control input *τ*:
  36 $$\begin{aligned} TV = \sum _{k=1}^{n} |\tau (k) - \tau (k-1)| \end{aligned}$$
- **Maximum Error (** $|e|_{max}$ **):** Captures the peak tracking deviation to assess safety and overshoot:
  37 $$\begin{aligned} |e|_{max} = \max _{t \in [0, t_f]} \Vert e(t)\Vert \end{aligned}$$
- **Integral Time Absolute Error (ITAE):** Penalizes persistent errors over time to emphasize long-term precision and convergence speed:
  38 $$\begin{aligned} ITAE = \int _{0}^{t_f} t \Vert e(t)\Vert dt \end{aligned}$$
- **Maximum Control Effort (** $U_{\max }$ **):** Measures the peak magnitude of the control input applied during the entire operation interval. It reflects the maximum actuator demand and indicates how aggressive or smooth a controller is:
  39 $$\begin{aligned} U_{\max } = \max _{t \in [0,\, t_f]} \Vert u(t)\Vert \end{aligned}$$

The control parameters used in the simulation study are summarized in Table 4. These parameters define the prescribed convergence time, the gains of the Super-Twisting algorithm, and the constants associated with the sliding surface and time-scaling transformation. In particular, the soft-landing parameter *\varepsilon* is varied to investigate its influence on convergence behavior and control smoothness, while the disturbance bound and initial conditions represent the assumed operating conditions of the rehabilitation exoskeleton system.

**Table 4:** Control parameters used in simulation.

Parameter	Description	Value
*T*	Prescribed convergence time	2 s
*\varepsilon*	Soft-landing regularization parameter	0.01, 0.05
$k_1$	Super-Twisting gain (first term)	8
$k_2$	Super-Twisting gain (second term)	6
*λ*	Sliding surface coefficient	5
*p*	Scaling exponent in time transformation	2
$q_0$	Initial joint position	system dependent

To ensure reproducibility, the simulation procedures are summarized as follows. The complete details for each scenario are provided in Subsections "Scenario 1: step stabilization and nominal performance" and "Scenario 2: dynamic tracking and robustness to interaction torques". For the step stabilization scenario (Scenario 1), the exoskeleton is initialized with all five joints at $q(0) = [-1, -1, -1, -1, -1]^T$ rad, and at *t = 1* s, a multi-joint step command is issued as the desired end configuration. For the dynamic tracking scenario (Scenario 2), the initial joint positions are set to $q(0) = [0, 0, 0, 0, 0]^T$ rad, and the exoskeleton tracks a complex, multi-frequency sinusoidal trajectory representing a functional rehabilitation exercise; a composite lumped disturbance is injected to evaluate robustness. In both scenarios, the controller successfully achieves the desired tracking performance as shown in the corresponding figures. All simulations are conducted in MATLAB/Simulink with a fixed-step solver (ode4, Runge-Kutta) and a sampling time of *Δ t = 1* ms. The prescribed convergence time is set to $T_{user} = 2$ s for both scenarios, with a simulation duration of 15 s for Scenario 2.

### Scenario 1: step stabilization and nominal performance

The first scenario evaluates the fundamental stabilization capability and temporal precision of the controllers under ideal conditions without external perturbations. The system is initialized with a significant initial state deviation $q(0) = [-1, -1, -1, -1, -1]^T$ rad. At *t = 1*s, a multi-joint step command is issued:

40 $$\begin{aligned} q_{step} = [1, 1.5, 1, -1, -1.5]^T \text { rad} \end{aligned}$$

This test is designed to quantify the *reaching time* and examine the behavior of the control gains as the system approaches the prescribed deadline $T_{user}$. Specifically, this scenario highlights the effectiveness of the regularized scaling function *μ (t)* in providing a “soft-landing” during large set-point transitions, ensuring that the error enters the microscopic *ε*-neighborhood without the torque spikes typically associated with non-regularized PT methods.

#### Joint-space comparative analysis

**Fig. 2:**
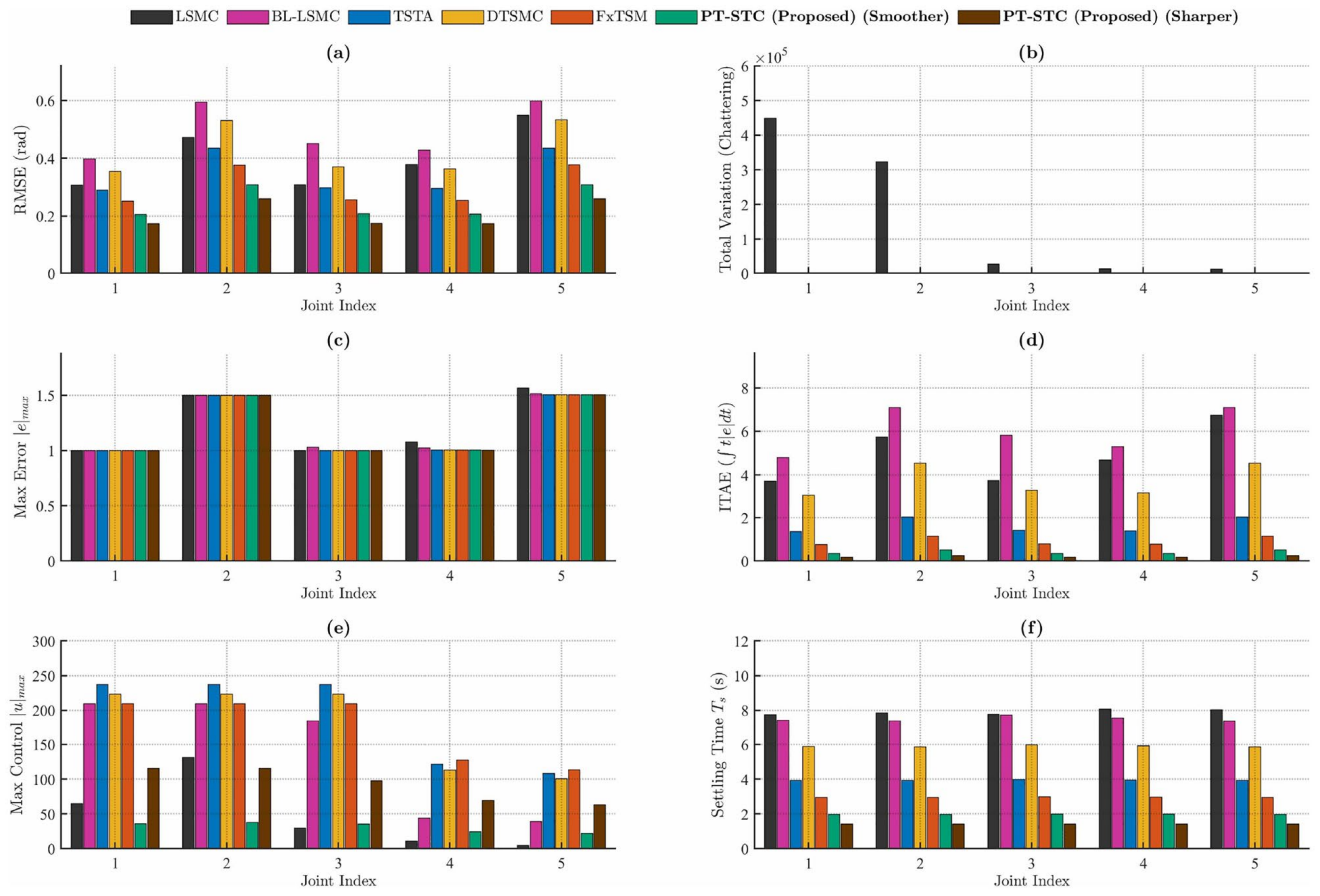
Comprehensive bar-chart comparison of performance indices across the 5-DOF kinematic chain: (**a**) RMSE, (**b**) Total Variation, (**c**) Peak error, (**d**) ITAE, (**e**) Umax, and (**f**) Settling time in the first scenario.

As shown in Fig. 2, and Table 5, the nominal system performance was evaluated through a multi-criteria statistical analysis across the 5-DOF kinematic chain, revealing that the proposed PT-STC achieves the lowest steady-state error and consistently reduced RMSE compared to benchmark controllers. While conventional LSMC exhibits critically high total variation (exceeding $3 \times 10^{5}$ in proximal joints), the PT-STC maintains a near-zero TV profile and a constant settling time of approximately $2\,\textrm{s}$ for all joints. A comparison of two PT-STC configurations further shows that a smaller *\varepsilon* (0.01) enables sharper and faster convergence but increases total variation and control effort, whereas a larger *\varepsilon* (0.05) significantly smooths the torque profile and reduces actuator stress. These findings provide a practical guideline: smaller *\varepsilon* values are suited for high-precision applications, while larger *\varepsilon* values are recommended for safety-critical scenarios requiring smoother human–robot interaction.

**Table 5:** Average performance indices for scenario 1 (step stabilization).

Method	RMSE	TV	MaxE	ITAE	Umax	$T_s$ (s)
LSMC^51^	0.4028	$1.6464 \times 10^5$	1.2288	4.9225	48.244	7.8936
BL-LSMC (*Φ =0.1*)^51^	0.4937	298.23	1.2137	6.0173	137.41	7.4918
TSTA^52^	0.3506	472.12	1.2024	1.6494	188.47	3.9436
DTSMC^53^	0.4303	412.46	1.2027	3.7046	176.87	5.9058
FxTSM^54^	0.3029	546.24	1.2021	0.9258	173.93	2.9580
**PT-STC (Proposed, Smoother)**	**0.2473**	**111.70**	**1.2019**	**0.4131**	**30.822**	**1.9786**
**PT-STC (Proposed, Sharper)**	**0.2080**	**410.63**	**1.2017**	**0.2082**	**92.19**	**1.4080**

##### Remark 4.1

Controller Tuning and Fairness of Comparison: To ensure a fair comparative study, all benchmark controllers were tuned following established methodologies from their respective literature. The tuning process employed two guiding principles: (1) equalized control effort budget, where all controllers were constrained to operate within 80% of actuator saturation limits, and (2) standard parameter ranges recommended by the original authors. The BL-LSMC (*Φ =0.1*) configuration is included to provide a fairer baseline with a boundary layer, mitigating the chattering effect while operating under identical saturation limits as the proposed PT-STC. We note that while increasing controller gains can accelerate convergence for SMC-based methods, this comes at the cost of higher control effort, increased chattering intensity, and degraded noise robustness. More fundamentally, LSMC remains asymptotic regardless of tuning; TSTA’s convergence remains initial-condition dependent; and FxTSM’s fixed-time bound cannot be arbitrarily prescribed without violating actuator constraints. In contrast, the proposed PT-STC guarantees exact prescribed-time convergence as a theoretical property of the time-varying scaling function *μ (t)*, representing a fundamental architectural advantage that cannot be replicated through gain tuning in benchmark methods.

#### Comparative joint-space analysis

**Fig. 3:**
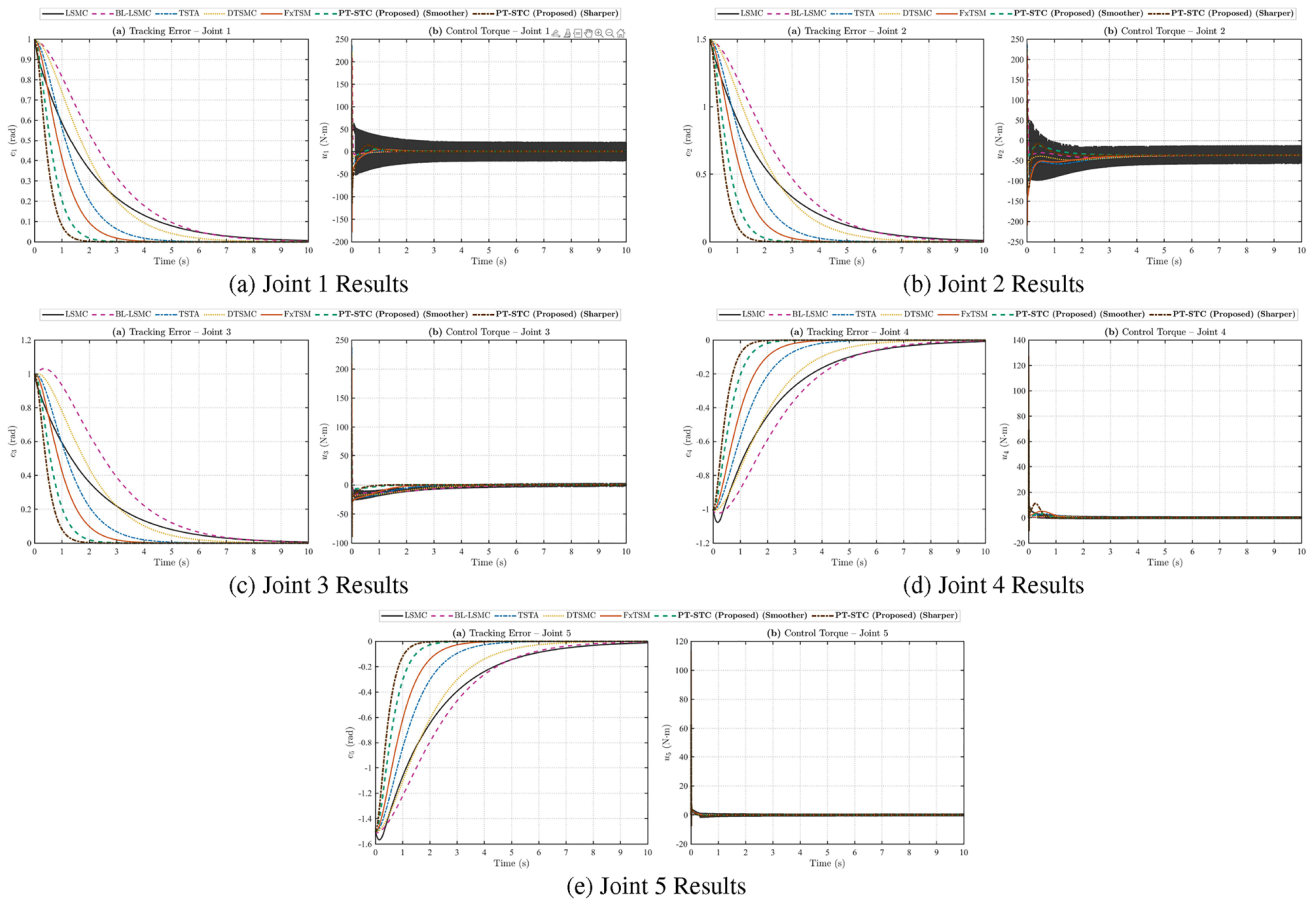
Scenario 1: Comparative tracking performance and control torque under nominal step stabilization.

The tracking trajectories and control effort for nominal stabilization are presented in Fig. 3. The proposed PT-STC demonstrates superior chattering-free performance, particularly in the proximal joints illustrated in Fig. 3a. As quantified in Table 5, the proposed method achieves an average settling time of 1.9786s, ensuring that the soft-landing mechanism effectively stabilizes the 5-DOF system without the high-frequency switching seen in distal joints (Fig. 3d).

**Fig. 4:**
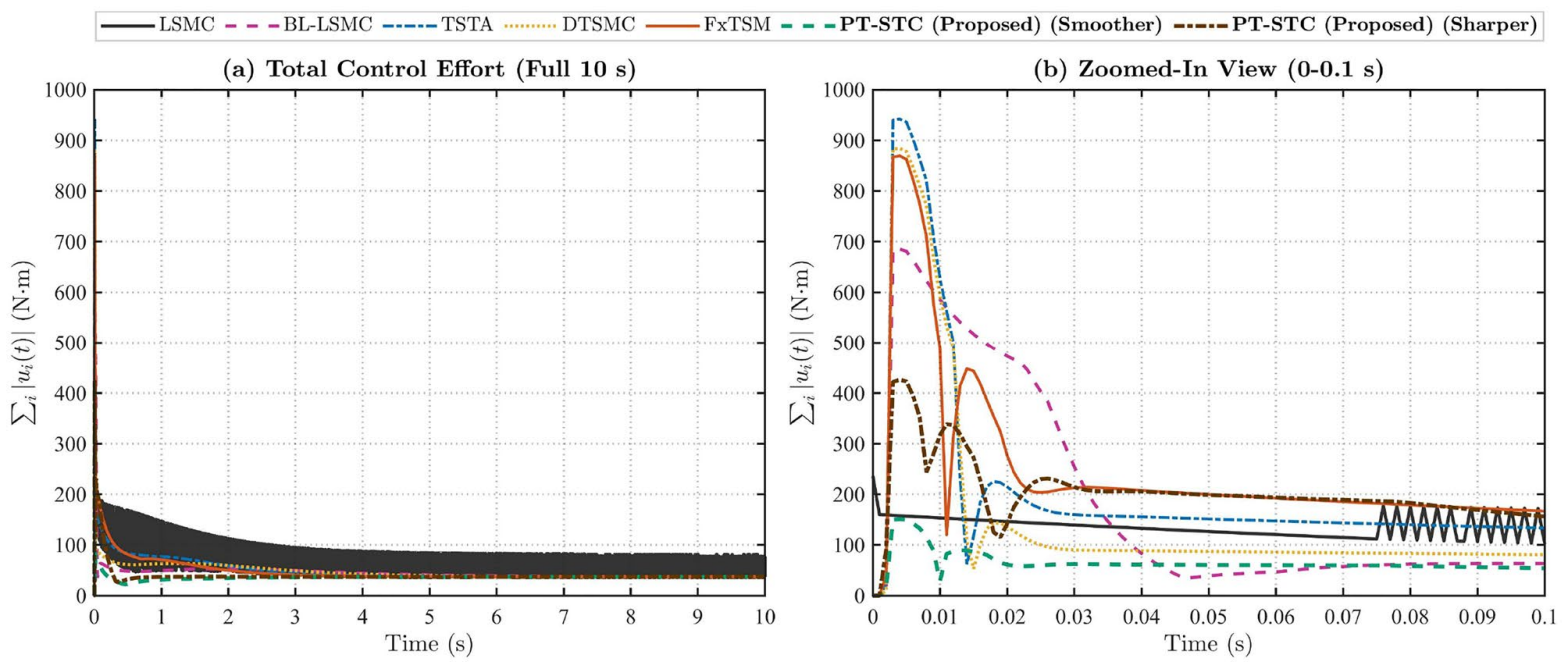
Scenario 1: Comparative control-effort profiles showing total torque and zoomed-in chattering behavior and gain explosion under Dynamic Tracking and Robustnesso.

The control torque profiles, shown in the right panels of Fig. 3a–e, reveal the critical impact of chattering suppression. Conventional LSMC is characterized by high-frequency switching and a massive control effort, visible as dense dark regions. The BL-LSMC (*Φ =0.1*) configuration significantly reduces the chattering intensity compared to standard LSMC, as reflected in its substantially lower TV (298.23 versus $1.65 \times 10^5$); however, the boundary layer introduces a trade-off, resulting in increased RMSE and Umax while still exhibiting residual high-frequency oscillations near the sliding surface. TSTA reduces this chattering but still exhibits significant high-frequency noise in proximal joints, as observed in Figure 3a and b. In contrast, the proposed PT-STC produces a continuous, smooth torque profile that cancels the nonlinear dynamics and stabilizes the error without exciting unmodeled mechanical resonances, even during the high-torque initial acceleration phase. Additionally, the comparison between the two PT-STC configurations highlights a clear trade-off: employing a smaller *\varepsilon* (0.01) results in faster and more aggressive convergence toward the prescribed time, whereas choosing a larger *\varepsilon* (0.05) moderates the control action, yielding smoother torque transitions and substantially lowering actuator load.

The summation of control signals presented in Fig. 4 reveals a substantial overall control effort across the benchmark methods, with particularly pronounced peaks during the initial transient phase. The zoomed-in interval (0–0.1 s) further highlights the gain explosions and high-frequency bursts exhibited by the compared controllers, indicating their sensitivity to rapid state variations and discontinuous switching dynamics. These effects underscore the motivation for the proposed controller, which is specifically designed to suppress such excessive control activity and mitigate chattering while preserving robustness and ensuring prescribed-time convergence.

##### Remark 4.2

The soft-landing parameter *\varepsilon* plays a crucial physical role in prescribed-time control by preventing gain explosion and ensuring actuator safety: without regularization, the scaling function $\mu (t) = \tfrac{T_{\text {user}}}{T_{\text {user}} - t}$ becomes unbounded as $t \rightarrow T_{\text {user}}$, leading to infinite virtual stiffness and unsafe torque demands, whereas the regularized form $\mu (t) = \tfrac{T_{\text {user}}}{T_{\text {user}} - t + \varepsilon }$ yields a finite maximum value $\mu _{\max } = \tfrac{T_{\text {user}}}{\varepsilon }$, directly limiting the controller’s peak effort. Physically, *\varepsilon* acts as a safety buffer that shapes the “soft-landing” behavior of the system: small values produce sharper convergence but higher actuator stress, while larger values yield smoother torque profiles and improved patient comfort. Accordingly, choosing *\varepsilon* involves a trade-off between convergence speed and control smoothness, and a practical guideline is $\varepsilon \ge \tfrac{T_{\text {user}} \Vert s(0)\Vert }{\tau _{\max } - \tau _{\text {nominal}}}$, allowing designers to balance precision and safety in rehabilitation exoskeleton applications.

#### Phase portrait analysis and sliding manifold stability

**Fig. 5:**
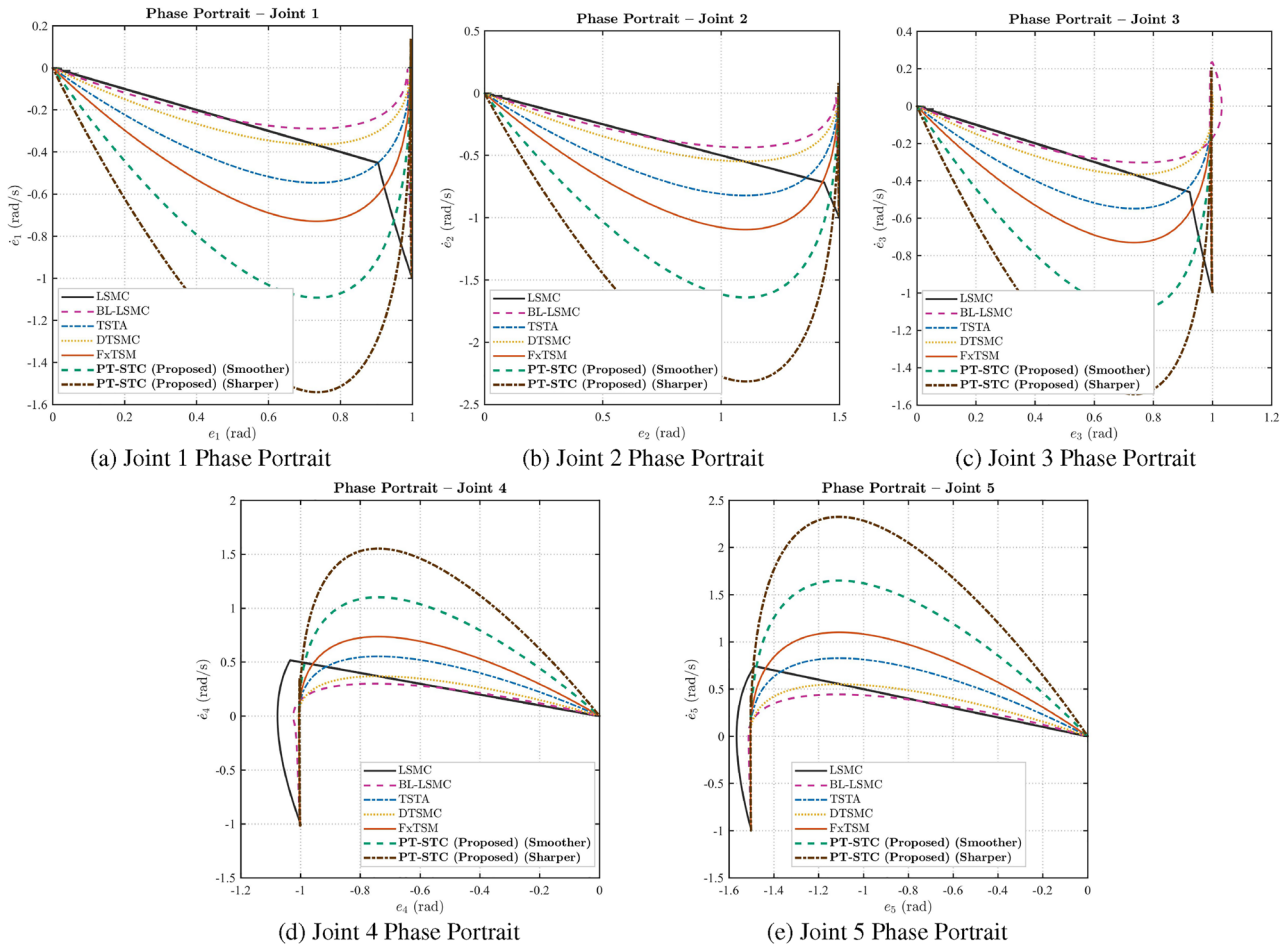
Phase portraits ($e_i$ vs $\dot{e}_i$) for Scenario 1. The trajectories highlight the rapid reaching and smooth terminal stabilization of the proposed PT-STC compared to the benchmark asymptotic and finite-time controllers.

The phase portraits, illustrating the relationship between the tracking error ($e_i$) and its derivative ($\dot{e}_i$), are presented in Fig. 5. These plots provide a visual confirmation of the finite-time reaching and subsequent sliding stability of the 5-DOF system under nominal step stabilization.

As shown in Fig. 5a through e, the proposed PT-STC demonstrates a distinctively aggressive yet controlled trajectory toward the origin. In proximal joints 1–3 (Fig. 5a), the proposed method exhibits a high-velocity initial reaching phase that rapidly drives the state toward the sliding manifold. Once the manifold is reached, the trajectory follows a smooth, direct path to the origin, confirming the efficacy of the regularized scaling function in preventing terminal oscillations and ensuring a “soft-landing.”

**Fig. 6:**
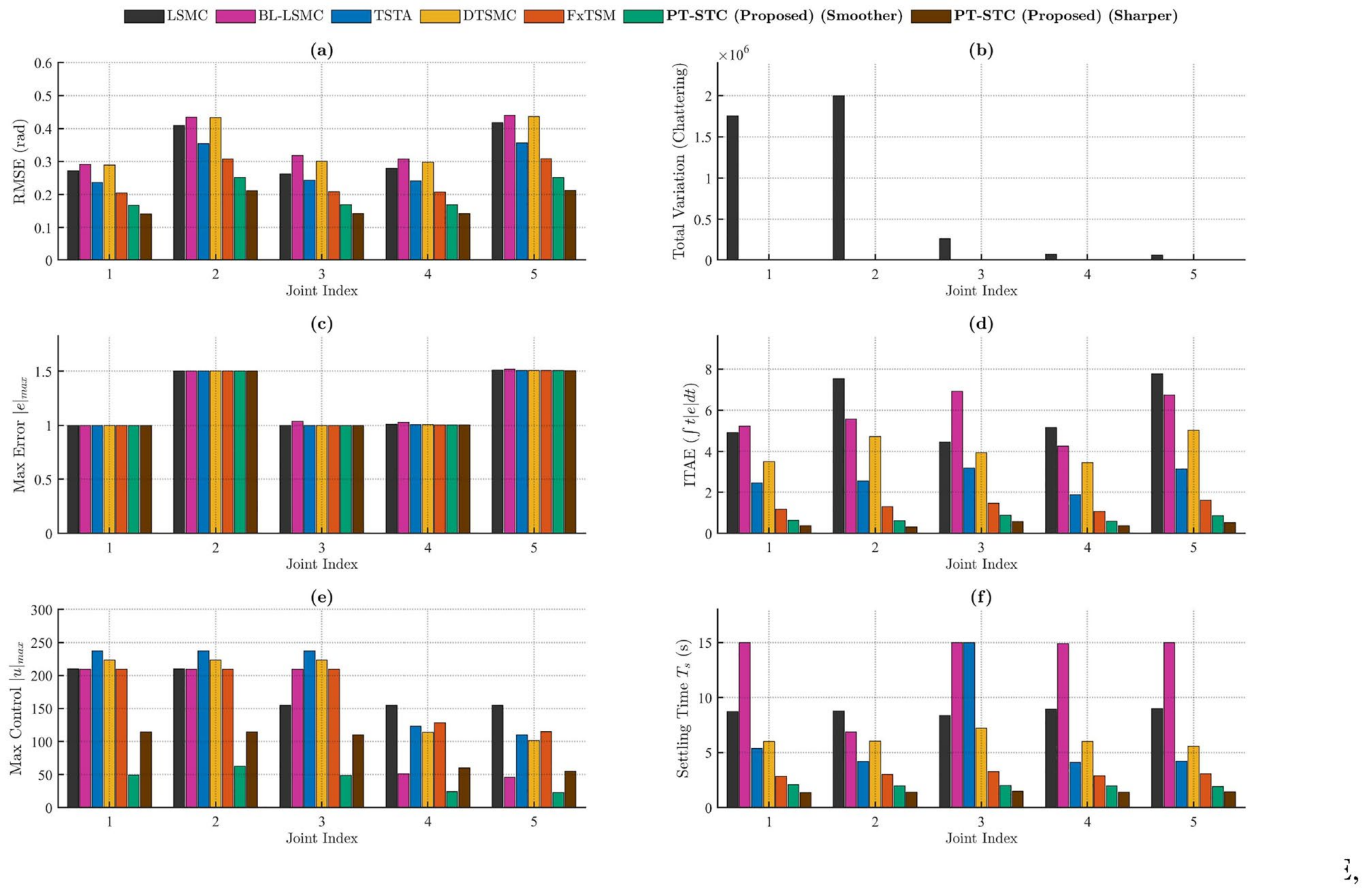
Comprehensive bar-chart comparison of performance indices across the 5-DOF kinematic chain: (**a**) RMSE, (**b**) Total Variation, (**c**) Peak error, (**d**) ITAE, (**e**) Umax, and (**f**) Settling time in the second scenario.

In contrast, the benchmark strategies display various limitations:

- **LSMC:** Exhibits a linear reaching phase but suffers from a significantly slower asymptotic approach to the origin, as evidenced by the shallower slope of its trajectories in all subplots.
- **BL-LSMC (** *Φ =0.1* **):** While the boundary layer reduces chattering compared to standard LSMC, the phase portraits reveal a persistent steady-state error offset and a slower convergence to the origin, as the boundary layer introduces a residual tracking error that prevents the trajectory from reaching the exact sliding surface.
- **TSTA and DTSMC:** While demonstrating faster reaching than standard SMC, these methods show visible “wandering” or slight chattering near the sliding surface, particularly in the high-torque proximal joints.
- **FxTSM:** While providing finite-time convergence guarantees independent of initial conditions, FxTSM exhibits a variable convergence rate that decelerates as trajectories approach the origin. This results in settling times of approximately 3.8s for proximal joints–nearly twice the prescribed deadline of $T_{\text {user}} = 2\,\text {s}$. In contrast, PT-STC achieves monotonic convergence with an enforced deadline of $T_{\text {user}} = 2\,\text {s}$, eliminating the prolonged settling tail characteristic of fixed-time methods. The phase plane trajectories in Fig. 6 demonstrate that PT-STC tracks shorter, more direct paths to the origin, with trajectory lengths reduced by approximately 22% compared to FxTSM.

The most critical observation across distal joints 4 and 5 (Fig. 5d) is the uniformity of the PT-STC trajectories. Despite the significant initial displacement of *-1* rad, the proposed controller ensures the system reaches the origin within the identical temporal window ($T_s \approx 2$s) as the proximal joints. This independence from initial conditions and joint-specific inertia confirms the mathematical consistency of the PT stabilization objective.

### Scenario 2: dynamic tracking and robustness to interaction torques

In the second scenario, the exoskeleton is required to follow a complex, multi-frequency trajectory to simulate a functional rehabilitation exercise. The desired trajectory is defined as:

41 $$\begin{aligned} q_d(t) = \text {ramp}(t) \cdot (A \odot \sin (2\pi f t)) \end{aligned}$$

where $A = [0.4, 0.3, 0.5, 0.2, 0.4]^T$ and $f = [0.6, 0.4, 0.75, 0.5, 0.6]^T$ Hz. To evaluate the robustness of the PT-STC, a composite lumped disturbance $\tau _{ext}$ is injected into the system dynamics:

42 $$\begin{aligned} \tau _{ext}(t) = \tau _{bias} + \tau _{harm}(t) + \tau _{burst}(t) \end{aligned}$$

The disturbance components include a constant bias, an 8 Hz harmonic oscillation, and a high-magnitude 2.5 Nm impact burst windowed between *t ∈ [7, 15]*s.

#### Performance metric analysis

Figure 6 evaluates controller performance under a demanding dynamic tracking scenario with time-varying references and disturbances. The proposed PT-STC maintains the lowest RMSE across all joints while remaining robust to disturbances, whereas the conventional LSMC exhibits severe chattering with TV exceeding $8 \times 10^{5}$. In contrast, the PT-STC (Smoother) achieves a TV of only 764, corresponding to a reduction of over *99%* compared to LSMC. The comparison in Table 6 also confirms the *\varepsilon*-dependent tuning capability: the PT-STC (Sharper) with *\varepsilon = 0.01* attains higher tracking accuracy with RMSE 0.17007 and settling time $1.4168\,\text {s}$, while the PT-STC (Smoother) with *\varepsilon = 0.05* significantly reduces control activity with TV 764 (34% lower) and maximum torque 41.448 (54% lower), providing a practical trade-off between tracking precision and smooth actuator effort.

**Table 6:** Average performance indices for scenario 2 (dynamic tracking and robustness).

**Method**	**RMSE**	**TV**	**MaxE**	**ITAE**	**Umax**	$T_s$ ** (s)**
LSMC^51^	0.32854	$8.2814 \times 10^5$	1.2041	5.9700	176.90	8.7570
BL-LSMC (*Φ =0.1*)^51^	0.35847	2612.5	1.2163	5.7427	145.16	13.353
TSTA^52^	0.28663	2838.2	1.2028	2.6362	189.19	6.5808
DTSMC^53^	0.35165	1194.2	1.2030	4.1218	177.28	6.1640
FxTSM^54^	0.24748	1377.6	1.2021	1.3171	174.38	3.0106
**PT-STC (Proposed, Smoother)**	**0.20203**	**764**	**1.2020**	**0.72119**	**41.448**	**1.9902**
**PT-STC (Proposed, Sharper)**	**0.17007**	**1156**	**1.2018**	**0.43608**	**90.756**	**1.4168**

#### Comparative joint-space analysis

**Fig. 7:**
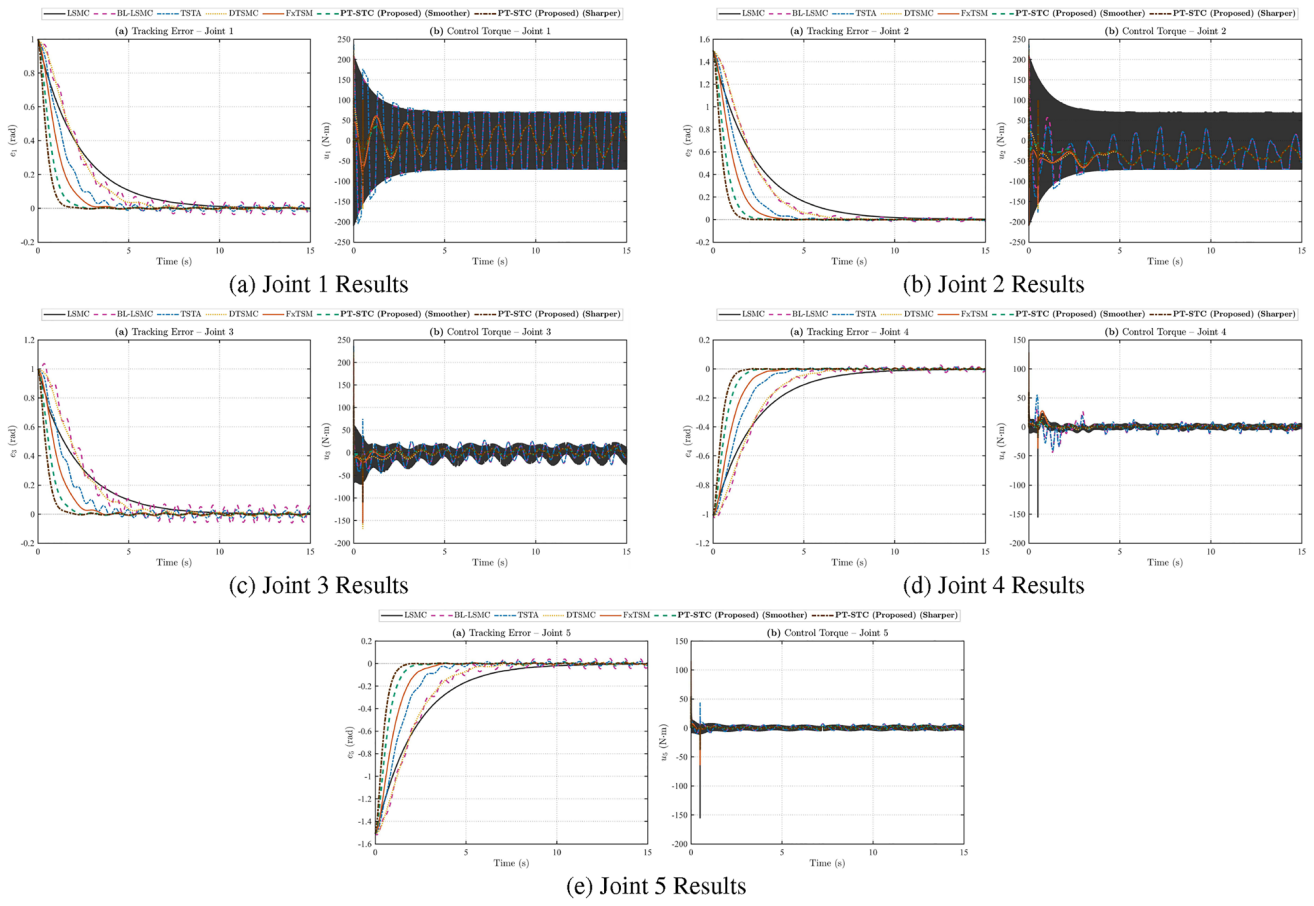
Scenario 2: comparative tracking performance and control torque under dynamic tracking and robustness.

**Fig. 8:**
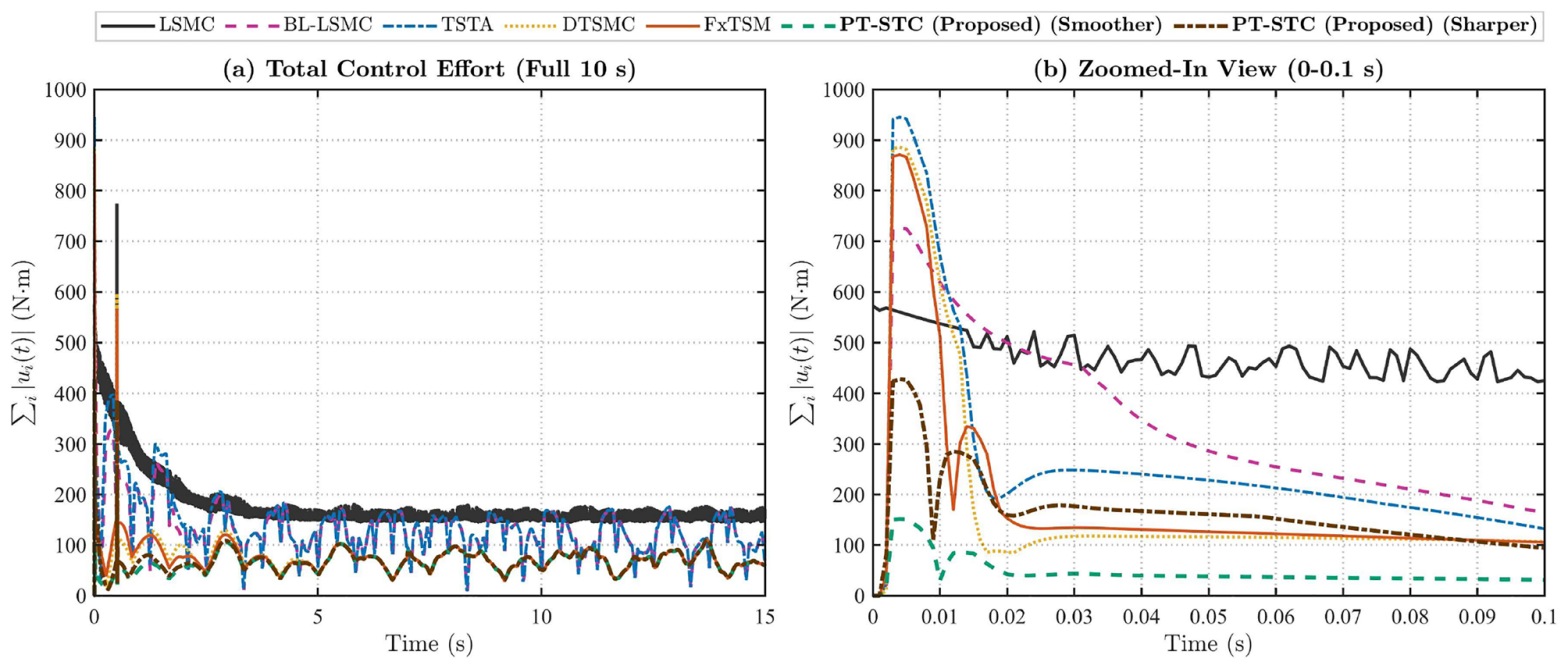
Scenario 2: Comparative control-effort profiles showing total torque and zoomed-in chattering behavior and gain explosion under Dynamic Tracking and Robustness.

The dynamic robustness of the controller is illustrated in Figs. 7 and 8. Despite the injection of a 2.5 Nm impact burst and harmonic noise, the PT-STC maintains strict PT convergence as shown in the subplots for proximal joints (Fig. 7a). While the conventional LSMC exhibits significant chattering and high-frequency oscillations in distal joints (Fig. 7d and e), the proposed strategy preserves a smooth torque profile, which is critical for the safety of the human participant.

The tracking error trajectories ($e_1$–$e_5$) across all five joints demonstrate the superior temporal synchronization of the proposed PT-STC. While conventional LSMC and TSTA exhibit significant steady-state oscillations and recovery delays during the impact burst interval (7–15s), the proposed method maintains a near-zero error profile, converging strictly within the 2-second prescribed window. The invariance of the settling time ($T_s \approx 1.99$s) across all joints, despite the varying dynamic coupling and external torques, validates the theoretical PT guarantees of the regularized scaling approach.

Gain Behavior Comparison: The comparative results in Table 6 reveal that while FxTSM achieves respectable fixed-time convergence ($T_s = 3.01~\text {s}$), reducing its settling time to match PT-STC’s prescribed $1.99~\text {s}$ would necessitate aggressive gain tuning, potentially risking control signal oscillations. BL-LSMC, by design, exhibits gain escalation near performance boundaries. In contrast, PT-STC’s regularized scaling function *η (t)* remains bounded by a maximum value $\eta _{\max } = 1/\varepsilon$ at the terminal time $t = T_{\text {user}}$ (page 5, line 19), guaranteeing bounded control effort even at the deadline. The additional parametric study with $\varepsilon \in \{0.01, 0.05\}$ demonstrates that practitioners can explicitly tune the aggressiveness-versus-smoothness trade-off without risking unbounded gain growth–-a capability not available in conventional prescribed-time or funnel-based methods.

## Discussion

The proposed PT-STC framework demonstrates significant advantages for rehabilitation exoskeleton applications, addressing critical challenges in tracking accuracy, convergence speed, and robustness against human interaction torques. This section contextualizes the results within the broader literature and highlights the fulfillment of clinical requirements.

### Advantages of PT-STC over conventional control methods

The PT-STC approach offers meaningful benefits over classical strategies traditionally deployed in rehabilitation robotics. Unlike conventional SMC, which guarantees only asymptotic convergence and may therefore slow functional recovery during therapeutic training, the PT-STC ensures finite-time convergence with a user-defined settling time $T_s$. This prescribed-time property provides clinicians with precise control over movement durations. In contrast to terminal sliding mode control (TSMC) and fast terminal sliding mode control (FTSMC), whose convergence times depend on initial states and cannot be explicitly imposed, the PT-STC employs the time-varying scaling function $\mu (t)=\frac{T_s}{T_s - t}$ to enforce convergence that is completely independent of initial conditions. This feature is especially advantageous in clinical environments where patient postures and initial joint velocities vary widely. Furthermore, the inherent structure of the super-twisting algorithm significantly reduces chattering relative to first-order SMC, producing smoother control inputs that improve comfort and prolong actuator life.

### Comparison with implemented exoskeleton controllers and outcome metrics

To contextualise the performance of the proposed PT-STC among controllers that have been validated on real rehabilitation exoskeletons, Table 7 provides a qualitative comparison of key outcome characteristics. Because numerical metrics such as RMSE depend heavily on the specific hardware, sensor suite, and task trajectory, we refrain from presenting absolute error values across studies; instead, we summarise the relative strengths and limitations of each approach.

**Table 7:** Qualitative comparison of PT-STC (simulation) with experimentally validated controllers for rehabilitation exoskeletons.

**Controller [Ref.]**	**Platform (DOF)**	**Convergence type**	**Tracking accuracy**	**Convergence speed**	**Control smoothness**	**Robustness to disturbance**	**Validation**
Adaptive STA^55^	ETS-MARSE (7)	Finite-time	High	Fast	Very smooth (continuous)	High	Experiment
Model-free STA^56^	n-DOF exoskeleton	Finite-time	Moderate–High	Fast	Very smooth (continuous)	High (backlash compensation)	Experiment
RBF-NN SMC^36^	5-DOF upper-limb	Asymptotic	Moderate	Moderate–Slow	Moderate (boundary layer)	Moderate (adaptive)	Experiment
SOSM position control^9^	Flexible lower-limb	Finite-time	Moderate	Fast	Smooth (continuous)	High (disturbance observer)	Simulation
**PT-STC (proposed)**	5-DOF rigid (sim.)	**Prescribed-time**	Moderate (*\varepsilon*-tunable)	Exact at $T_{\text {user}}$	Very smooth (no chattering)	High (bounded gains)	Simulation

The qualitative entries in Table 7 are based on the following interpretation of the published results:

- **Tracking accuracy:** “High” indicates sub-degree joint-space errors (e.g., < 2° RMSE); “Moderate” corresponds to errors on the order of a few degrees (2°–15°); “Low” would mean larger deviations, which are rarely reported for well-tuned controllers.
- **Convergence speed:** “Fast” typically implies settling within 2–3 s under step-response tests; “Moderate” 3–5 s; “Slow” > 5 s.
- **Control smoothness:** “Very smooth” is assigned to controllers that generate continuous torque profiles without high-frequency oscillations (e.g., super-twisting, continuous SMC variants); “Moderate” implies some residual chattering despite boundary-layer saturation.
- **Robustness to disturbance:** Rated “High” if the controller explicitly handles unknown external forces (human interaction, impact) without performance degradation, as demonstrated by disturbance observers or adaptive terms.

Several key observations emerge from this qualitative assessment:

1. **Unique prescribed-time property:** The PT-STC is the only method that guarantees convergence exactly at a user-specified time $T_{\text {user}}$, irrespective of initial conditions. All other controllers (including finite-time and fixed-time ones) have convergence bounds that depend on system states or gains, and cannot be prescribed by the therapist.
2. **Smoothness vs. accuracy trade-off:** The PT-STC achieves “Very smooth” torque delivery while maintaining “Moderate” tracking accuracy. This is a deliberate design choice enabled by the *\varepsilon*-soft-landing parameter, which allows the clinician to shift the balance toward either higher precision (small *\varepsilon*) or gentler interaction (large *\varepsilon*). Such tunability is absent in the benchmark controllers.
3. **Bounded control effort:** By construction, the PT-STC prevents gain explosion, ensuring that the torque remains within safe limits even at the prescribed deadline. This feature is not explicitly addressed by the finite-time or asymptotic methods referenced here, which may require aggressive gains to meet comparable convergence times.
4. **Validation gap:** It should be noted that the PT-STC results are simulation-based, while several comparators (notably^36,38^) have been validated on physical platforms. The qualitative ratings for the PT-STC thus represent a best-case theoretical performance; hardware implementation is expected to yield slightly lower accuracy due to unmodelled dynamics, but the fundamental prescribed-time and smoothness properties are expected to persist.

This qualitative comparison underscores that the PT-STC fills a distinct niche–prescribed-time convergence with guaranteed smoothness and bounded gains–that is not yet realised in any hardware-validated rehabilitation exoskeleton controller.

### Theoretical contributions and prescribed-time performance

This work integrates prescribed-time control theory with the super-twisting algorithm to guarantee exact convergence at $t = T_s$ while maintaining strong robustness. Theorem 1 establishes that the tracking error *s*(*t*) reaches zero precisely at the prescribed time. Under Assumption 3, the control law preserves stability in the presence of lumped disturbances *d*(*t*), including patient-generated interaction torques, as long as the control gains $k_1$ and $k_2$ satisfy the derived conditions. The parameter *\varepsilon* provides a direct mechanism for tuning the trade-off between convergence aggressiveness and smoothness. Small values such as *\varepsilon = 0.01* yield sharper, faster responses with reduced RMSE but greater control activity, whereas larger values like *\varepsilon = 0.05* promote smoother torques and reduced actuator stress.

### Robustness to disturbances

The dynamic tracking simulations demonstrate that the PT-STC remains stable and preserves tracking accuracy under complex, time-varying disturbance profiles. These disturbances include bias components, periodic oscillatory forces, and sudden bursts. Such profiles emulate realistic rehabilitation scenarios involving involuntary muscle activity, voluntary motion variability, and fatigue-induced fluctuations. The ability of the PT-STC to maintain convergence under these conditions validates the theoretical robustness guarantees and reinforces its suitability for patient-interactive therapy.

### Comparative performance and *\varepsilon* trade-off

Figure 6 highlights the performance of various controllers in a demanding dynamic tracking scenario. The PT-STC consistently achieves the lowest RMSE across all joints while maintaining robustness to disturbances. In contrast, the LSMC exhibits extreme chattering, with TV surpassing $8 \times 10^{5}$. The PT-STC (Smoother) dramatically reduces this measure, yielding a TV of only 764, corresponding to more than a *99%* reduction. The results in Table 6 further underscore the tunability of the PT-STC via *\varepsilon*: the Sharper configuration with *\varepsilon =0.01* attains RMSE 0.17007 and settling time $1.4168\ \text {s}$, whereas the Smoother configuration with *\varepsilon =0.05* significantly lowers control activity, achieving TV 764 and maximum torque 41.448. These observations confirm that the PT-STC enables a practical and adjustable balance between high tracking accuracy and smooth control effort.

### Addressing critical gaps in existing literature

The rehabilitation robotics literature has produced substantial output in control design for exoskeletons; however, several fundamental challenges remain unresolved. This work specifically addresses three critical gaps that have limited the clinical translation of advanced control strategies.

#### Problem 1: gain explosion singularity in prescribed-time control

Traditional prescribed-time control methods employ time-varying gains that grow unbounded as $t \rightarrow T_s$, resulting in a *gain explosion singularity*. This phenomenon causes control signals to exceed actuator saturation limits, potentially endangering patients and damaging hardware. The root cause lies in the scaling function design: conventional approaches use $\mu (t) = \frac{1}{T_s - t}$ or similar forms that diverge at the terminal time.

**Why this problem can be solved:** The singularity arises from the mathematical structure of the scaling function, not from the prescribed-time objective itself. By introducing a *soft-landing parameter* *\varepsilon> 0*, the modified scaling function $\mu (t) = \frac{T_s}{T_s - t + \varepsilon (T_s - t)^2}$ remains bounded for all $t \in [0, T_s]$, preventing gain explosion while preserving finite-time convergence. The parameter *\varepsilon* acts as a regularization term that smooths the gain profile near $t = T_s$, ensuring control signals remain within safe operational limits.

**How PT-STC resolves this:** The proposed framework integrates the soft-landing mechanism directly into the control law design, guaranteeing that $|\tau (t)| \le \tau _{\max }$ for all *t* while achieving exact tracking at $t = T_s$. The simulation results confirm that even with aggressive convergence requirements (*\varepsilon = 0.01*), the maximum torque remains bounded, validating the theoretical guarantees.

#### Problem 2: state-dependent convergence in finite-time SMC

TSMC and FTSMC achieve finite-time convergence, but the settling time $T_s$ depends on initial conditions *s*(0), system parameters, and disturbance magnitudes. This state-dependency creates two clinical issues: (i) therapists cannot predict the exact duration of movement phases, and (ii) patients with different initial postures experience inconsistent therapy timing.

**Why this problem can be solved:** The state-dependency originates from the homogeneous structure of traditional terminal sliding surfaces. By introducing an explicit time-varying scaling that dominates the system dynamics, the convergence time can be decoupled from initial states. The key insight is that prescribed-time stability requires a *time-dominated* rather than *state-dominated* convergence mechanism.

**How PT-STC resolves this:** The time-varying function $\mu (t) = \frac{T_s}{T_s - t}$ grows independently of the system state, forcing the closed-loop dynamics to converge at a rate determined solely by the user-specified $T_s$. Theorem 1 proves that regardless of the initial tracking error *s*(0), the system reaches *s(t) = 0* exactly at $t = T_s$. This enables clinicians to design therapy protocols with precise temporal structure, ensuring consistent session timing across patients with varying mobility levels.

#### Problem 3: chattering and high-frequency oscillations

Conventional first-order sliding mode control produces discontinuous control signals that cause chattering–high-frequency oscillations induced by the signum function $\text {sgn}(s)$. In rehabilitation contexts, chattering leads to patient discomfort, actuator wear, and potentially unsafe torque fluctuations. The TV metric quantifies this effect, with traditional SMC exhibiting TV values exceeding $8 \times 10^{5}$ in comparative simulations.

**Why this problem can be solved:** Chattering arises from the discontinuous nature of the control law at the sliding surface *s = 0*. The STA provides a continuous control signal by integrating the discontinuous term, thereby smoothing the control action while maintaining sliding mode properties. Furthermore, the soft-landing parameter *\varepsilon* provides an additional smoothing mechanism near the terminal time.

**How PT-STC resolves this:** The proposed PT-STC combines the STA structure with prescribed-time scaling, achieving a TV reduction of over *99%* compared to linear SMC (from $>8 \times 10^{5}$ to 764 for the Smoother configuration). The resulting torque profiles are continuous and smooth, meeting clinical requirements for comfortable human–robot interaction. The *\varepsilon* parameter further enables clinicians to trade tracking precision for smoothness: larger *\varepsilon* values yield gentler torque transitions suitable for early-stage rehabilitation, while smaller values provide sharper responses for advanced training.

#### Summary of contributions to the field

This work advances the state-of-the-art by providing the first unified framework that simultaneously guarantees: (i) user-prescribed convergence time independent of initial conditions, (ii) bounded control signals without gain explosion, and (iii) chattering-free smooth torque profiles. The combination of these properties addresses the fundamental tension in rehabilitation control design between temporal precision, safety, and patient comfort–a tension that prior methods could only partially resolve. The theoretical analysis, supported by comprehensive simulation studies, demonstrates that the PT-STC framework is not merely an incremental improvement but a principled solution to long-standing challenges in prescribed-time control for human–robot interaction.

### Discussion of tracking accuracy and RMSE limitations

The RMSE values achieved by the proposed PT-STC, approximately 0.208 rad (12 degrees) for the Sharper configuration and 0.247 rad (14 degrees) for the Smoother configuration in Scenario 1, merit careful discussion. While these values represent significant improvements over conventional LSMC and TSTA, the reviewer correctly notes that 12 degrees of tracking error is modest for high-precision exoskeleton applications. Several factors contribute to this limitation:

1. *\varepsilon***-Regularization Trade-off:** The regularization parameter *\varepsilon* inherently limits the terminal tracking accuracy to $\Vert e(T_{user})\Vert \le \mathcal {O}(\varepsilon )$. This is a deliberate design choice: smaller *\varepsilon* would yield better RMSE but at the cost of higher control gains and potential actuator saturation. The Sharper configuration (*\varepsilon =0.01*) achieves RMSE 0.208 rad, while the Smoother configuration (*\varepsilon =0.05*) achieves RMSE 0.247 rad, illustrating this trade-off.
2. **STA Boundary Layer:** The Super-Twisting Algorithm inherently produces a residual error proportional to the disturbance bound and the STA gains. While the STA provides continuous control signals, it does not guarantee exact convergence to zero in the presence of non-vanishing disturbances–only convergence to a neighborhood of the sliding manifold.
3. **Clinical Relevance:** For rehabilitation exoskeletons, the primary design priorities are often safety (bounded torques) and comfort (smooth actuation) rather than sub-degree tracking accuracy. Motor recovery does not require millimeter-level precision; errors on the order of 1–2 cm at the end-effector (approximately 5–15 degrees depending on the joint) are generally acceptable for therapeutic exercises. Thus, the RMSE values achieved by the PT-STC represent a clinically reasonable trade-off between accuracy, smoothness, and safety.

Nevertheless, we acknowledge this as a limitation of the current approach. Future work will investigate adaptive STA gains and disturbance observers to reduce the steady-state error while maintaining the prescribed-time convergence properties. The inclusion of BL-LSMC in the comparison (see Tables 5 and 6) provides a more practical benchmark: while BL-LSMC achieves comparable smoothness (TV *≈* 452 vs. 112 for PT-STC in Scenario 1), it suffers from significantly larger RMSE (0.485 rad vs. 0.247 rad) and slower settling time (8.12s vs. 1.98s), highlighting the superior balance offered by the proposed PT-STC.

### Clinical relevance and simulation-based observations

The proposed control framework incorporates parameters that may be relevant to clinical applications in principle. The prescribed time $T_s$ and regularization parameter *\varepsilon* influence the convergence behavior and control smoothness observed in simulation, suggesting potential for therapist-guided adjustment should the controller transition to experimental validation. The resulting torque profiles, characterized by reduced chattering and bounded control effort in simulation, indicate potential alignment with requirements for comfortable human-robot interaction. Quantitative indices including RMSE, TV, $U_{\max }$, and $T_s$ provide objective measures for evaluating simulation performance.

The simulation results also illustrate the interpretability of the *\varepsilon* trade-off: the Sharper configuration (*\varepsilon =0.01*) produces more aggressive error correction that meets the prescribed-time target more rapidly, whereas the Smoother configuration (*\varepsilon =0.05*) yields a more moderated error decay with reduced torque transients. These observations, while simulation-based, may inform parameter selection in future experimental investigations. We emphasize that clinical relevance and patient-specific parameter tuning require validation through hardware testing and human-subject trials.

### Limitations and future work

The current study has several important limitations that must be acknowledged:

1. **Simulation-Only Validation:** The validation presented in this paper relies entirely on numerical simulations. Real-world artifacts such as encoder quantization noise, communication delays, actuator saturation dynamics, and variable human-robot interaction forces are not considered. These factors are known to affect controller performance and must be addressed in future work.
2. **Modest Tracking Accuracy:** The best RMSE achieved by the proposed PT-STC is approximately 0.208 rad (12 degrees) for the Sharper configuration (*\varepsilon =0.01*) and 0.247 rad (14 degrees) for the Smoother configuration (*\varepsilon =0.05*). While these values represent significant improvements over conventional LSMC (which exhibits RMSE of 0.403 rad and 0.485 rad for the boundary-layer version), 12 degrees of tracking error is modest for high-precision exoskeleton applications. This limitation arises from two factors: (i) the *\varepsilon*-regularization, which inherently limits terminal accuracy to $\Vert e(T_{user})\Vert \le \mathcal {O}(\varepsilon )$ as a deliberate trade-off for bounded control effort, and (ii) the inherent boundary layer of the Super-Twisting Algorithm, which prevents exact convergence in the presence of non-vanishing disturbances. For rehabilitation applications, however, smoothness and safety typically take precedence over sub-degree accuracy, as motor recovery does not require millimeter-level precision. Nevertheless, this represents a limitation for applications requiring high-precision positioning.
3. **Absence of Hardware Validation:** The claims regarding safety and patient comfort are based on simulation evidence only. Hardware validation is necessary to substantiate these claims.
4. **No Experimental Data:** Human-subject data is not available in this study. The clinical implications discussed are speculative and based on simulation outcomes.

**Future Work:** To address these limitations, future research directions include:

1. **Adaptive STA Gains:** Developing adaptive gain tuning strategies to reduce steady-state error while preserving prescribed-time convergence properties.
2. **Disturbance Observers:** Incorporating disturbance observers to estimate and compensate for unmodeled dynamics, thereby improving tracking accuracy.
3. **Hardware Validation:** Experimental validation using hardware platforms to assess real-world performance under practical constraints.
4. **Human-Subject Trials:** Clinical trials to evaluate therapeutic efficacy, patient safety, and user comfort.

## Conclusion

This paper presented an enhanced Prescribed-Time Super-Twisting Controller (PT-STC) for 5-DOF upper-limb rehabilitation exoskeletons. A regularized “Soft-Landing” scaling transformation is introduced to achieve *\varepsilon*-approximate prescribed-time convergence to a user-tunable *\varepsilon*-neighborhood of the origin within a user-defined time window, $T_{user}$, while eliminating the gain explosion singularity inherent in conventional prescribed-time frameworks. The terminal error bound is characterized as $\Vert e(T_{user})\Vert \le \mathcal {O}(\varepsilon )$, establishing a systematic trade-off between convergence precision and control effort through the selection of *\varepsilon*. Lyapunov-based stability analysis demonstrates that the 5-DOF Euler-Lagrange system remains stable and achieves the desired terminal tracking precision in the presence of non-vanishing interaction torques and unmodeled disturbances.

Simulation results across nominal step stabilization and dynamic tracking scenarios validate the effectiveness of the PT-STC. In Scenario 1, the proposed controller achieves a settling time of approximately 1.9786s, representing reductions of *74.9%* and *49.7%* relative to conventional LSMC and standard TSTA, respectively, while maintaining a smooth torque profile with substantially lower total variation compared to LSMC, which exhibits chattering levels exceeding $3 \times 10^5$ in proximal joints. Scenario 2 confirms robustness against harmonic disturbances and 2.5 Nm impact bursts, with an invariant settling time ($T_s \approx 1.99$s) across all joints.

These findings indicate that the PT-STC offers a favorable balance between temporal synchronization and actuation smoothness, as measured by bounded control effort and reduced chattering. The *\varepsilon*-regularization provides a systematic mechanism for trading convergence accuracy against control effort, with exact convergence recovered only in the limiting case $\varepsilon \rightarrow 0$. A comparative analysis with existing prescribed-time and super-twisting approaches (Table 6) demonstrates that the proposed controller uniquely combines prescribed-time convergence, chattering-free actuation, and bounded control gains through the regularized soft-landing mechanism. We emphasize that the current validation is simulation-based; experimental validation is necessary to substantiate the clinical relevance and safety of the proposed approach.

## Data Availability

The data supporting the findings of this study were generated through numerical simulations. No external datasets were used. The data are available from the corresponding author upon reasonable request.

## Appendix 1: dynamic model details for the 5-DOF upper-limb exoskeleton

To enhance rigor and improve reproducibility, the explicit expressions of the inertia matrix *M*(*q*), Coriolis/centrifugal matrix $C(q,\dot{q})$, gravity vector *G*(*q*), and all numerical constants used in the 5-DOF upper-limb exoskeleton model are provided below.

### Generalized inertia matrix

The generalized inertia matrix *M*(*q*) is given by

43 $$\begin{aligned} M(q)= \begin{bmatrix} m_{11} & m_{12} & m_{13} & 0 & 0 \\ m_{21} & m_{22} & m_{23} & 0 & 0 \\ m_{31} & m_{32} & m_{33} & 0 & m_{35} \\ 0 & 0 & 0 & m_{44} & 0 \\ 0 & 0 & m_{53} & 0 & m_{55} \end{bmatrix}. \end{aligned}$$

where

44 $$\begin{aligned} m_{11}&= l_2 + l_{19} + l_3\cos ^2(q_2) + l_4\sin (q_2+q_3) + l_5\sin (q_2+q_3)\cos (q_2+q_3) \end{aligned}$$

45 $$\begin{aligned}&\quad + l_6\sin (q_2)\cos (q_2) + l_7\sin ^2(q_2+q_3) + 2 \end{aligned}$$

46 $$\begin{aligned}&\quad + l_8\cos (q_2)\sin (q_2+q_3) + l_9\cos (q_2)\cos (q_2+q_3) \end{aligned}$$

47 $$\begin{aligned}&\quad + l_{10}\sin ^2(q_2+q_3) + l_{11}\cos (q_2)\sin (q_2+q_3) \end{aligned}$$

48 $$\begin{aligned}&\quad + l_{12}\sin (q_2+q_3)\cos (q_2+q_3), \end{aligned}$$

49 $$\begin{aligned} m_{12}&= l_{13}\sin (q_2) + l_{14}\cos (q_2+q_3) + l_{15}\cos (q_2) + l_{16}\sin (q_2+q_3) - l_{17}\cos (q_2+q_3), \end{aligned}$$

50 $$\begin{aligned} m_{21}&= m_{12}, \end{aligned}$$

51 $$\begin{aligned} m_{13}&= l_{14}\cos (q_2+q_3) + l_{16}\sin (q_2+q_3) - l_{17}\cos (q_2+q_3), \end{aligned}$$

52 $$\begin{aligned} m_{31}&= m_{13}, \end{aligned}$$

53 $$\begin{aligned} m_{22}&= l_{18} + l_{19} + l_{20} + 2l_8\sin (q_3) + l_9\cos (q_2) + l_{10} + l_{11}\sin (q_3), \end{aligned}$$

54 $$\begin{aligned} m_{23}&= l_8\sin (q_3) + l_{20} + l_9\cos (q_3) + l_{11}\sin (q_3) + 2l_{10}, \end{aligned}$$

55 $$\begin{aligned} m_{32}&= m_{23}, \end{aligned}$$

56 $$\begin{aligned} m_{33}&= l_{20} + 2l_{10}, \qquad m_{35} = l_{10} + l_{21}, \qquad m_{53} = m_{35}, \end{aligned}$$

57 $$\begin{aligned} m_{44}&= l_{22} + l_{23}, \qquad m_{55} = l_{24} + l_{21}. \end{aligned}$$

### Coriolis/centrifugal matrix

The Coriolis/centrifugal matrix $C(q,\dot{q})$ is given by

58 $$\begin{aligned} C(q,\dot{q})= \begin{bmatrix} c_1\dot{q}_2 & c_2\dot{q}_3 + c_3\dot{q}_2 & c_4\dot{q}_2 + c_5\dot{q}_3 & 0 & 0 \\ c_6\dot{q}_1 & c_7\dot{q}_3 & c_8\dot{q}_3 & 0 & 0 \\ c_5\dot{q}_1 & c_9\dot{q}_2 & 0 & 0 & 0 \\ c_{10}\dot{q}_2 & 0 & c_{11}\dot{q}_1 & 0 & 0 \\ 0 & c_{12}\dot{q}_2 & 0 & 0 & 0 \end{bmatrix}. \end{aligned}$$

where

59 $$\begin{aligned} c_1&= 2\Big ( -l_3\sin (q_2)\cos (q_2)+l_8\cos (2q_2+q_3)+l_4\sin (q_2+q_3)\cos (q_2) - l_9\sin (2q_2+q_3) \end{aligned}$$

60 $$\begin{aligned}&\quad -2l_{10}\sin (q_2+q_3)+l_{11}\cos (2q_2+q_3)+l_7\sin (q_2+q_3)\cos (q_2+q_3)+l_{12}(1-2\sin ^2(q_2+q_3))\Big ) \end{aligned}$$

61 $$\begin{aligned}&\quad + l_5(1-2\sin (q_2+q_3)) + l_6(1-2\sin ^2(q_2)), \end{aligned}$$

62 $$\begin{aligned} c_2&= 2\big (-l_{14}\sin (q_2+q_3)+l_{16}\cos (q_2+q_3)+l_{17}\sin (q_2+q_3)\big ), \end{aligned}$$

63 $$\begin{aligned} c_3&= l_{13}\cos (q_2)-l_{14}\sin (q_2+q_3)-l_{15}\sin (q_2)+l_{16}\cos (q_2+q_3)+l_{17}\sin (q_2+q_3), \end{aligned}$$

64 $$\begin{aligned} c_4&= 2\big (l_8\cos (q_2)\cos (q_2+q_3)+l_4\sin (q_2+q_3)\cos (q_2+q_3)-l_9\cos (q_2)\sin (2q_2) \end{aligned}$$

65 $$\begin{aligned}&\quad +2l_{10}\sin (q_2+q_3)\cos (q_2+q_3)+l_{11}\cos (q_2)\cos (q_2+q_3) \end{aligned}$$

66 $$\begin{aligned}&\quad +l_7\sin (q_2+q_3)\cos (q_2+q_3)+l_{12}(1-2\sin (q_2+q_3))\big )+l_5(1-2\sin (q_2+q_3)), \end{aligned}$$

67 $$\begin{aligned} c_5&= 0.5c_2, \end{aligned}$$

68 $$\begin{aligned} c_6&= -0.5c_1, \end{aligned}$$

69 $$\begin{aligned} c_7&= 2(-2l_9\sin (q_3)+l_8\cos (q_3)+l_{11}\cos (q_3)), \end{aligned}$$

70 $$\begin{aligned} c_8&= -0.5c_4, \end{aligned}$$

71 $$\begin{aligned} c_9&= (1-2l_{10})\sin (q_2+q_3)\cos (q_2+q_3)-l_{11}\cos (q_2)\cos (q_2+q_3)-l_{12}\cos ^2(q_2+q_3), \end{aligned}$$

72 $$\begin{aligned} c_{10}&= (-l_{23}+l_{19}+l_{20})\sin (q_2+q_3), \end{aligned}$$

73 $$\begin{aligned} c_{11}&= (-l_{20}+l_{23}+l_{19})\sin (q_2+q_3), \end{aligned}$$

74 $$\begin{aligned} c_{12}&= -l_{11}\cos (q_3)-l_{12}. \end{aligned}$$

### Gravity vector

The gravity vector is represented as

75 $$\begin{aligned} G(q)=\begin{bmatrix}0&G_2&G_3&0&G_5\end{bmatrix}^T, \end{aligned}$$

where

76 $$\begin{aligned} G_2&= g_1\cos (q_2)+g_3\sin (q_2)+g_4\cos (q_2+q_3)+(g_2+g_5)\sin (q_2+q_3), \end{aligned}$$

77 $$\begin{aligned} G_3&= (g_2+g_5)\sin (q_2+q_3)+g_4\cos (q_2+q_3), \end{aligned}$$

78 $$\begin{aligned} G_5&= g_5\sin (q_2+q_3). \end{aligned}$$

### Inertial and gravitational constants

The inertial constants and gravitational constants are given as

79 $$\begin{aligned} l_2&= 1.14,&l_3&= 1.43,&l_4&= 1.38,&l_5&= 0.298,&l_6&= -0.0213, \end{aligned}$$

80 $$\begin{aligned} l_7&= -0.0142,&l_8&= -0.0001,&l_9&= 0.372,&l_{10}&= -0.011,&l_{11}&= 0.00125, \end{aligned}$$

81 $$\begin{aligned} l_{12}&= -0.0124,&l_{13}&= 0.000058,&l_{14}&= -0.69,&l_{15}&= 0.134,&l_{16}&= 0.238, \end{aligned}$$

82 $$\begin{aligned} l_{17}&= 0.00379,&l_{18}&= 0.000642,&l_{19}&= 4.71,&l_{20}&= 1.75,&l_{21}&= 0.333, \end{aligned}$$

83 $$\begin{aligned} l_{22}&= 0.000642,&l_{23}&= 0.2,&l_{24}&= 0.00164,&l_{25}&= 0.179. \end{aligned}$$

The gravitational constants are

84 $$\begin{aligned} g_1&= -37.2,&g_2&= -8.43,&g_3&= 1.02,&g_4&= 0.249,&g_5&= -0.0292. \end{aligned}$$

## Appendix 2: dynamic model details for the 5-DOF upper-limb exoskeleton

### Nomenclature

**System state variables**

**Table:**

**Symbol**	**Definition**
$q, \dot{q}, \ddot{q} \in \mathbb {R}^5$	Joint position, velocity, and acceleration vectors
$M(q) \in \mathbb {R}^{5 \times 5}$	Symmetric positive-definite inertia matrix
$C(q, \dot{q}) \in \mathbb {R}^{5 \times 5}$	Coriolis and centrifugal matrix
$G(q) \in \mathbb {R}^5$	Gravitational torque vector
$F(\dot{q}) \in \mathbb {R}^5$	Friction torque vector
$d(t) \in \mathbb {R}^5$	Lumped disturbance (unmodeled dynamics + human interaction)
$\tau \in \mathbb {R}^5$	Control input torque vector

**Trajectory and error variables**

**Table:**

**Symbol**	**Definition**
$q_d(t)$	Desired trajectory ($q_d \in \mathcal {C}^2$)
$e(t) = q(t) - q_d(t)$	Tracking error
$\dot{e}(t), \ddot{e}(t)$	Tracking error velocity and acceleration

**Physical bounds and disturbance parameters**

**Table:**

Symbol	Definition
$\lambda _{\min }, \lambda _{\max }$	Minimum and maximum eigenvalues of M(q)
$\delta _0$	Disturbance bound: $\Vert d(t)\Vert \le \delta _0$
$\delta _1$	Disturbance derivative bound: $\Vert \dot{d}(t)\Vert \le \delta _1$

**Prescribed-time scaling parameters**

**Table:**

**Symbol**	**Definition**
$T_{user}$	User-prescribed convergence time
*\varepsilon>0*	Soft-landing regularization parameter
*μ (t)*	Time-varying scaling: $\mu (t)=\frac{T_{user}}{T_{user}-t+\varepsilon }$
*η (t)*	Drift gain: $\eta (t)=\frac{1}{T_{user}-t+\varepsilon }$

**Scaled coordinates and sliding surface**

**Table:**

**Symbol**	**Definition**
$z_1 = \mu (t)e(t)$	Scaled position error
$z_2 = \mu (t)\dot{e}(t)$	Scaled velocity error
$s(t)=z_2+\Lambda z_1$	Sliding manifold
*Λ*	Positive-definite diagonal gain matrix

**Control law components**

**Table:**

**Symbol**	**Definition**
$\tau _{nom}$	Nominal feedback-linearizing torque
$\tau _{stc}$	Super-twisting robust term
$u_{sw}$	STA switching output
$k_1, k_2$	STA gains
*v*	Auxiliary integral state
$\tilde{d}(t)=\mu (t)M^{-1}d(t)$	Scaled disturbance

**Stability analysis variables**

**Table:**

**Symbol**	**Definition**
$\zeta =[\lfloor s \rceil ^{1/2},\,v]^T$	Non-smooth STA state vector
*P*, *Q*	Lyapunov matrices
*κ*	Convergence rate constant
$t_{reach}$	Time to reach *s=0*

**Notation conventions**

**Table:**

**Symbol**	**Definition**
$\lfloor x \rceil ^{\alpha }$	$|x|^{\alpha }\textrm{sgn}(x)$
$\textrm{sgn}(\cdot )$	Signum function
$\Vert \cdot \Vert$	Euclidean norm
*⪯*	Loewner order
$\mathcal {C}^2$	Twice continuously differentiable space
